# A New Class of Tunable Acid-Sensitive Linkers for Native Drug Release Based on the Trityl Protecting Group

**DOI:** 10.1021/acs.bioconjchem.2c00310

**Published:** 2022-08-18

**Authors:** Matt Timmers, Jimmy Weterings, Michiel van Geijn, Roel Bell, Peter E. Lenting, Cristianne J.F. Rijcken, Tina Vermonden, Wim E. Hennink, Rob M.J. Liskamp

**Affiliations:** † Cristal Therapeutics, Maastricht 6229 EV, The Netherlands; ‡ Department of Pharmaceutics, Utrecht Institute for Pharmaceutical Sciences, Utrecht University, Utrecht 3584 CG, The Netherlands; § Symeres, Nijmegen 6546 BB, The Netherlands; ∥ School of Chemistry, University of Glasgow, Glasgow G12 8QQ, U.K.; ⊥ Department of Biochemistry, Cardiovascular Research Institute Maastricht (CARIM), Maastricht University, Maastricht 6229 ER, The Netherlands

## Abstract

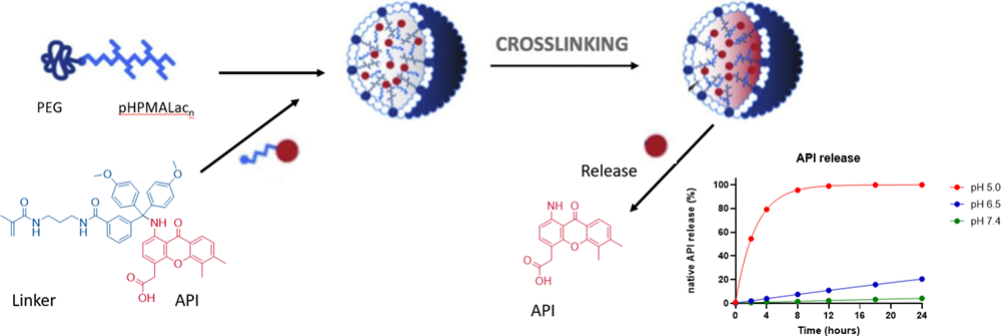

Core-cross-linked polymeric micelles (CCPMs) are a promising nanoparticle platform due to favorable properties such as their long circulation and tumor disposition exploiting the enhanced permeability and retention (EPR) effect. Sustained release of covalently linked drugs from the hydrophobic core of the CCPM can be achieved by a biodegradable linker that connects the drug and the core. This study investigates the suitability of trityl-based linkers for the design of acid-triggered native active pharmaceutical ingredient (API) release from CCPMs. Trityl linker derivatives with different substituent patterns were synthesized and conjugated to model API compounds such as DMXAA-amine, doxorubicin, and gemcitabine, and their release kinetics were studied. Hereafter, API release from CCPMs based on mPEG-b-pHPMAmLac block copolymers was investigated. Variation of the trityl substitution pattern showed tunability of the API release rate from the trityl-based linker with *t*_1/2_ varying from <1.0 to 5.0 h at pH 5.0 and *t*_1/2_ from 6.5 to >24 h at pH 7.4, all at 37 °C. A clear difference in release kinetics was found between gemcitabine and doxorubicin, with gemcitabine showing no detectable release for 72 h at pH 5.0 and doxorubicin showing a *t*_1/2_ of less than 1 h. Based on these findings, we show that the reaction mechanism of trityl deprotection plays an important role in the API release kinetics. The first step in this mechanism, which is protonation of the trityl-bound amine, is pK_a_-dependent, which explains the difference in release rate. In conclusion, acid-sensitive and tunable trityl linkers are highly promising for the design of linker–API conjugates and for their use in CCPMs.

## Introduction

Nanoparticulate drug delivery systems offer solutions to well-known challenges in chemotherapy such as therapeutic drug levels below the minimum effective concentration, poor pharmacokinetics, and unfavorable tissue distribution. Nanoparticles have been developed to achieve a better therapeutic efficacy with less side effects, thereby increasing the therapeutic index.^1−6^ These systems vary from lipid-based systems such as liposomes^7,8^ and metallic and inorganic nanoparticles^9−11^ to polymeric dendrimers and nanoparticles.^12−14^ Particularly, polymeric micelles based on amphiphilic block copolymers are attractive systems due to, among others, the tunability of their characteristics such as size, surface properties, drug loading, and release. Furthermore, by proper selection of the building blocks, systems with good cyto- and biocompatibility can be designed.^15−19^ Because polymeric micelles are dynamic systems and consequently encounter stability issues in particular in biological media, attention is given in recent years to core-cross-linked polymeric micelles (CCPMs), which have shown their potential in (pre)clinical studies.^20−25^ The hydrophobic core of CCPMs can be loaded with active pharmaceutical ingredients (APIs). A hydrophilic coat, mostly consisting of poly(ethylene glycol) (PEG), ensures colloidal stability and prolonged circulation time of these nanoparticles. A physically encapsulated API is not always sufficiently retained in the hydrophobic core of the CCPMs, which can be overcome by the covalent linkage of the API to the CCPM. Upon use of a biodegradable linker between the nanoparticle core and API, the release of native API can be governed by the degradation kinetics of the degradable bond between the API and linker attached to the hydrophobic micellar core. Native release refers to the released API not containing any residual molecular fragment as any modification to the API might impact its pharmacological and thus therapeutic activity.

**Figure 1:**
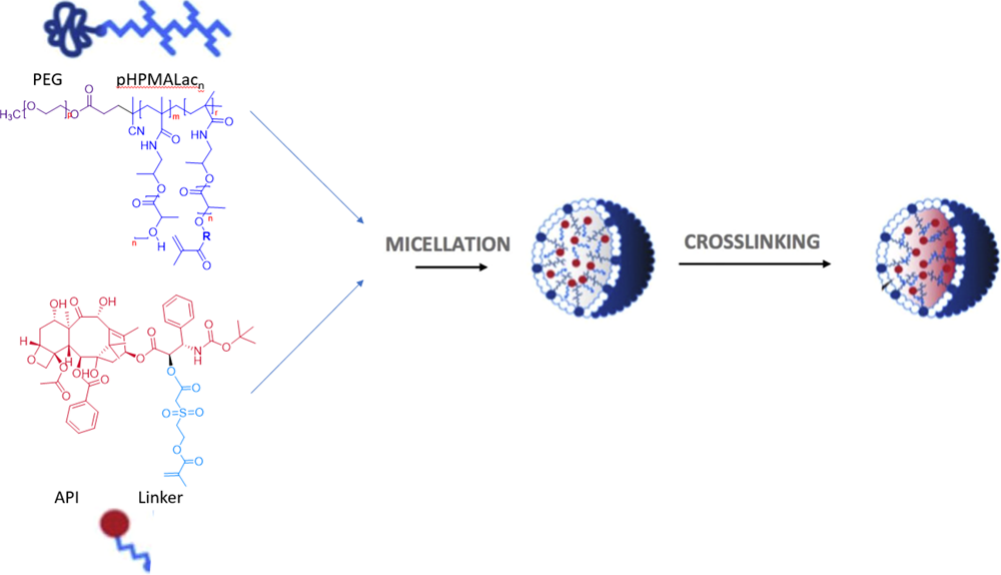
CCPMs based on mPEG-b-pHPMAmLac block copolymers and model API attached to a methacrylate linker. Upon micelle formation and free radical polymerization, the core of micelles is cross-linked simultaneously grafting the methacrylated API to the formed polymeric network.^22,27^ In the present study, three different model APIs (Figure 2) were coupled to the core of these CCPMs via a similar methodology.

In our research studies, CCPMs based on mPEG-b-pHPMAmLac block copolymers, which are partly functionalized with methacrylate moieties as shown in Figure 1, were investigated for their therapeutic potential.^20,26,27^ The amphiphilic block copolymers spontaneously form micelles in aqueous solution and APIs with a methacrylate-bearing linker can be loaded into the core. The micelles and APIs are then covalently cross-linked by free radical polymerization as previously shown.^26,28^ These CCPMs showed desirable effects in (pre)clinical settings,^29,30^ with prolonged circulation, reduced side effects, and fourfold higher tumor uptake as important benefits compared to free API. For an optimal therapeutic effect, the release should be minimal in the circulation after intravenous administration as this allows the CCPMs to exploit the enhanced permeability and retention (EPR) effect.^31−34^ Release should then preferably occur in the tumor microenvironment (TME).

Different triggers can be exploited to induce cleavage of the bond that connects the API with its carrier or destabilize the carrier, such as hydrolysis,^20^ enzymatic action,^35,36^ low redox potential,^37,38^ and pH changes.^39−41^ Trigger-sensitive release in the environment of interest is attractive as this can create a differentiation between release rates in healthy and pathological tissues. For example, acid-sensitive linkers are of interest for preferential release of chemotherapeutic APIs in the TME, which is often slightly acidic.^42−44^ Cellular internalization via endosomal/lysosomal uptake is expected of API carriers such as nanoparticles.^45−47^ These cellular compartments are known to have a low pH,^48,49^ facilitating a pH-triggered release of an API which can then cross the endosomal membrane.

Acid-sensitive API carrier systems containing hydrazone,^50−54^ acetal,^55,56^ orthoester,^57,58^ or imine^59−63^ moieties have been developed and investigated. These molecular moieties are not easily chemically modified, which limits the possibilities to adjust and tailor the release kinetics of APIs. Acid-sensitive release of protective groups is well documented,^64^ and it can provide inspiration to develop new generations of labile linkers for tailorable API release under slightly acidic conditions. Particularly, the triphenyl methyl (trityl) group is very attractive for this purpose. As early as in 1900, it was shown that the trityl group can form a stable radical.^65^ Similarly, the carbocation of the trityl group is very stable, which is explained by the resonance stabilization of the three aromatic rings. This stability of the trityl carbocation and therefore its facile formation led to many applications of the trityl group as an acid labile protecting group for use in the synthesis of relatively sensitive molecules such as peptides and carbohydrates^64,66^ as well as the use of the structurally closely related even more acid-sensitive dimethoxytrityl (DMTr) group in RNA and DNA synthesis.^67−70^

**Scheme 1:**
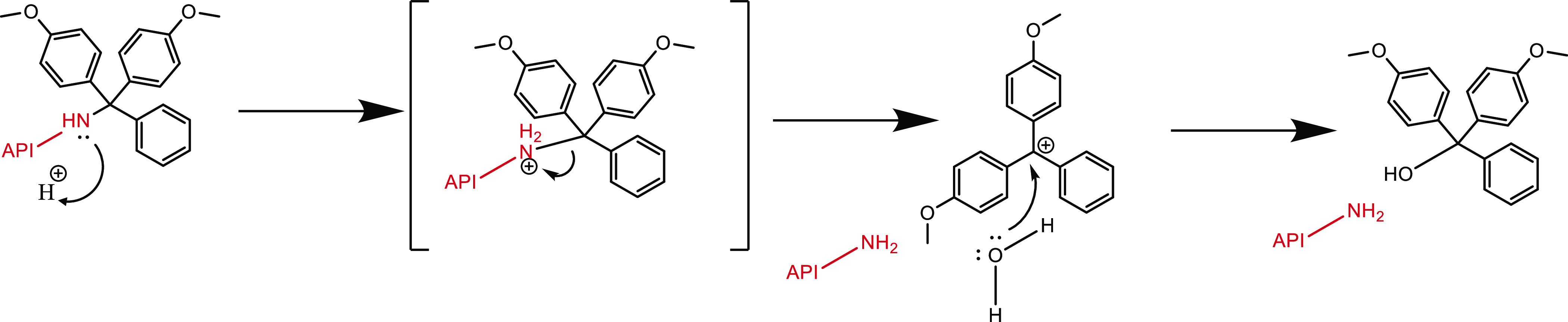
Acid-Catalyzed Hydrolytic Cleavage of the API–Trityl Conjugate Resulting in the Release of the Native API

Because of its relatively good stability, the (substituted) trityl carbocation is readily formed upon protonation under mildly acidic conditions (Scheme 1). The higher acid instability of the DMTr protecting group compared to the trityl protecting group hints at possibilities to tune the release kinetics of an API based on the substituents present on the trityl group. This could be expanded to the extent that a suitable trityl derivative could be developed for cleavage of an API in an acidic pH environment as is found in the endo/lysosomes (pH 4.5–5.0) or in the TME ranging from pH 6.3–7.0 in a relevant timeframe,^48,49^ with minimal release at a physiological pH of 7.4.

Seminal research by Patel et al. showed the influence of substituents on the acid instability of the trityl group.^71^ These substituents can either stabilize or destabilize the released carbocation, depending on the presence, number, and (*ortho*/*para*/*meta*) position of inductively or mesomerically electron-donating/withdrawing groups. This research showed the tunable release of the coupled difluorodeoxynucleoside compound. Subsequent research also showed the use of a (highly) substituted trityl group in an immunoconjugate, allowing for acid-sensitive release of the difluorodeoxynucleoside from an antibody.^72^ Shchepinov and Korshun expanded this methodology to a (substituted) trityl-containing linker in a hydrolyzable DNA–oligomer conjugate with cytotoxic payload.^73^ However, the precise release rate was not investigated.

**Figure 2:**
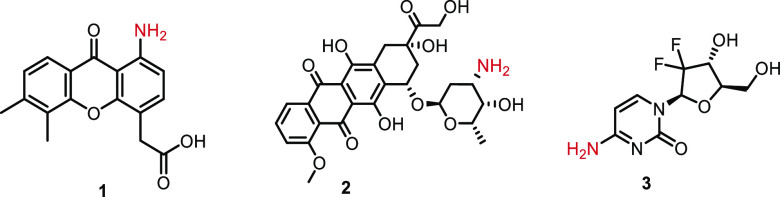
Structure of the model compounds investigated in this study: DMXAA-amine **1**, doxorubicin **2**, and gemcitabine **3**.

Clearly, the use of trityl functionalities has high potential in the design of drug carrier systems with tunable release kinetics. However, the effect of the characteristics of the conjugated API on the release kinetics has not yet been studied. The instability of the cleavable bond in an API–molecular construct is governed by both the API and the applied linker. Therefore, in the present study, we studied the dependency of the molecular characteristics of three selected APIs (structures shown in Figure 2) on their relative stability at neutral pH and acid-induced release from substituted trityl-containing linkers connected to a polymerizable moiety. Thus, various constructs were generated to investigate to what extent the release kinetics of an API–model linker construct can be modified. Next, these API–linkers were loaded into CCPMs. Release kinetics of the API from both the construct and CCPM were studied as a function of pH and compared to other trityl linkers bearing different substituents.

## Results and Discussion

The following API model compounds were selected: dimethyl xanthenone-acetic acid-C1 amine (DMXAA-amine) **1**, doxorubicin **2**, and gemcitabine **3** (Figure 2). These compounds are expected to have an improved therapeutical effect when incorporated in CCPMs due to prolonged circulation. The model compounds contain an amino functionality for attachment to a substituted trityl-containing linker and were chosen since they display differences in basicity, which might affect the release kinetics, that is, DMXAA-amine **1** contains an aromatic amine, doxorubicin **2** contains an aliphatic amine, and gemcitabine **3** contains an (hetero) aromatic amine. Therefore, the effect of the substitution pattern of the trityl linker on the release kinetics of each of these compounds was investigated under both neutral and slightly acidic conditions. Herein, the substituted trityl moiety was attached to the model compounds.

**Scheme 2:**
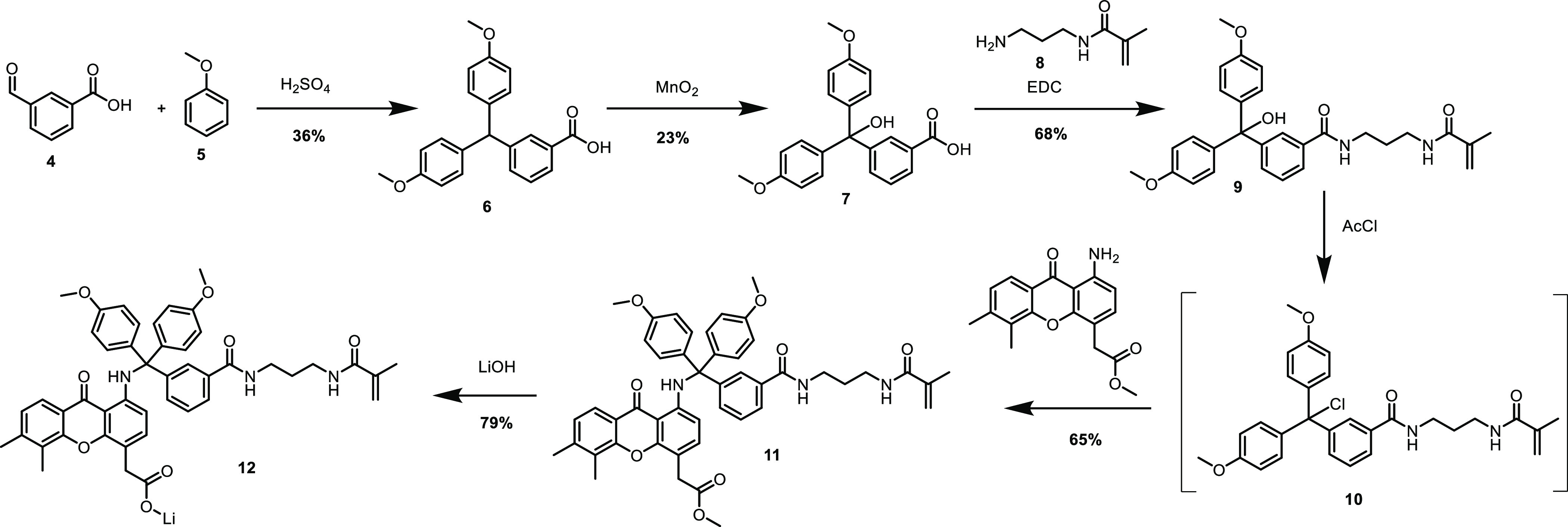
Synthesis Route of Trityl-DMXAA-Amine Construct **12**

For subsequent use in CCPMs, a polymerizable methacrylamide moiety was attached via a carboxylamide to the trityl group on the *meta* position, which is only a moderate electron-withdrawing group and therefore is expected to have a small impact on the stability of the trityl carbocation formed after cleavage of the bond between the API and the trityl linker. The synthesis route of the trityl-DMXAA-amine construct **12**, which is based on the DMTr group, is shown in Scheme 2. Starting from 3-formyl benzoic acid **4** and anisole **5**, a trityl moiety **6** was obtained, which was subsequently oxidized to trityl alcohol compound **7** using manganese dioxide. Using an EDC coupling method, the polymerizable methacrylamide part **8** was introduced, leading to a trityl alcohol construct **9**. The trityl chloride derivative **10** required for reaction with the amine moiety present on the model compound was prepared in situ using acetyl chloride (AcCl). Next, this trityl chloride derivative **10** was successfully coupled to the methyl ester of compound **1**, resulting in **11**. Saponification of **11** successfully gave the model compound–linker construct **12** as its lithium salt. Full synthesis details are provided in the Supporting Information (S2.1).

**Figure 3:**
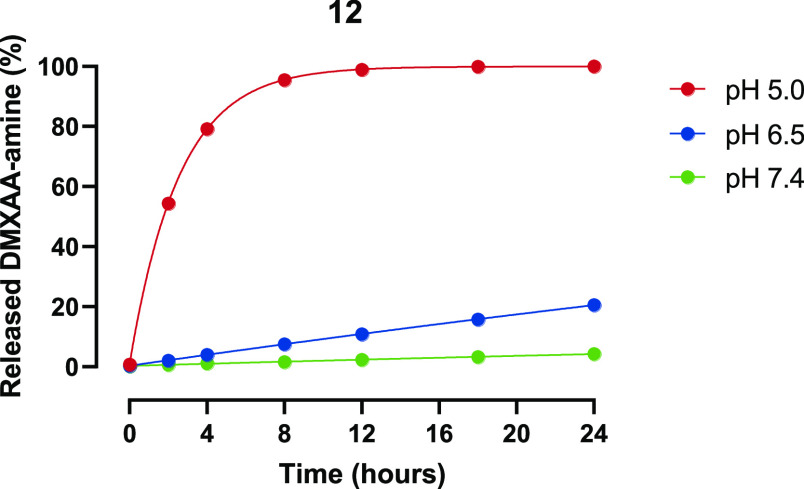
Measured release of native compound DMXAA-amine **1** from **12** at different pH values at 37 °C.

Release of DMXAA-amine **1** from the linker construct **12** (method described in S3.1) was measured at 37 °C for 24 h. At set time points, the concentration of the native compound **1** in a buffer of different pH values was measured by ultrahigh performance liquid chromatography (UHPLC) as shown in Figure 3. Hardly any release was observed at pH 7.4 over 24 h, while only ∼20% release was found at pH 6.5. As expected, a much faster release (100% within 12 h) was observed at a pH of 5.

**Figure 4:**
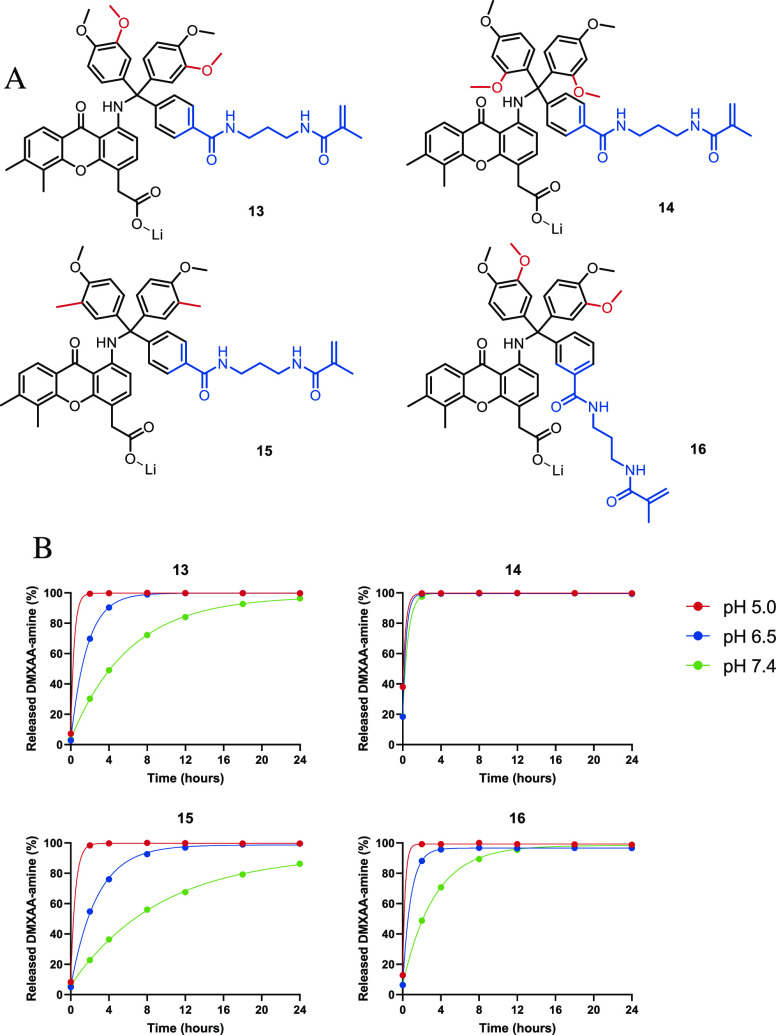
(A) Structures of trityl-DMXAA-amine derivatives **13**–**16** and (B) their corresponding release rates of native DMXAA-amine at different pH values and at 37 °C.

Additional trityl derivatives were synthesized that vary in number and position of methoxy and methyl substituents on the aromatic rings of the trityl functionality to investigate the impact of the substituents on the stability of the formed trityl carbocation and thereby the release rate. The variations in these, compounds **13–16**, are shown in Table 1 and final structures are shown in Figure 4A. Details of the synthesis procedures are provided in the Supporting Information (S2.2–2.5).

**Table 1:** Overview of Trityl Linker Derivatives **12**–**16**

#	para	meta	ortho	cross-linker position
**12**	OMe	H,H	H,H	meta
**13**	OMe	OMe, H	H,H	para
**14**	OMe	H,H	OMe, H	para
**15**	OMe	Me, H	H,H	para
**16**	OMe	OMe, H	H,H	meta

Figure 4B shows the release of native DMXAA-amine from derivatives **13**–**16** in a buffer with varying pH values and at 37 °C. The half-life values (*t*_1/2_) were determined from the slope of the first-order kinetic curves fitted through the release curve and are shown in Table 2. Details are provided in Section S3 of the Supporting Information.

**Table 2:** *t*_1/2_ Values (in h) of Trityl-DMXAA-Amine Constructs **12**–**16** at Different pH Values and at 37 °C (ND: Not Determined)

	*t*_1/2_ pH 5.0 (h)	*t*_1/2_ pH 6.5 (h)	*t*_1/2_ pH 7.4 (h)
**12**	1.8 ± 0.1	83 ± 27	ND
**13**	<1	1.2 ± 0.1	4.2 ± 0.2
**14**	<1	<1	<1
**15**	<1	1.9 ± 0.2	6.5 ± 0.5
**16**	<1	<1	2.3 ± 0.1

Table 2 shows that compound **14** with two electron-donating methoxy groups at the *ortho* and *para* position and the moderately electron-withdrawing linker at the *para* position gave rise to the fastest release kinetics with a *t*_1/2_ of <1 h at pH 7.4. This observation is in agreement with the expected fastest release as the methoxy groups in these positions donate electrons to the formed carbocation, favoring the release and formation of the stabilized carbocation. Compound **15**, having only one methoxy substituent and the less (inductively) donating methyl substituent in addition to the moderately electron-withdrawing substituent (carboxamide) containing a linker at the *para* position, showed a considerably slower release with a *t*_1/2_ of 6.5 h at pH 7.4. As expected, the methyl substituent at the *meta* position having minimal effect on the stability of the formed carbocation had no significant impact on the release rate. The methacrylamide cross-linking moiety at either *para* (**13**) or *meta* (**16**) position did not have a large impact on the release kinetics.

Although these *t*_1/2_ values of API–linker constructs give information regarding the trends of release rates, they may not be representative for the release of APIs from a CCPM. Therefore, CCPMs were prepared, purified, and characterized with model compound–linker constructs **12–16** incorporated, following a previously described procedure (see Supporting Information Section S4).^26^

**Figure 5:**
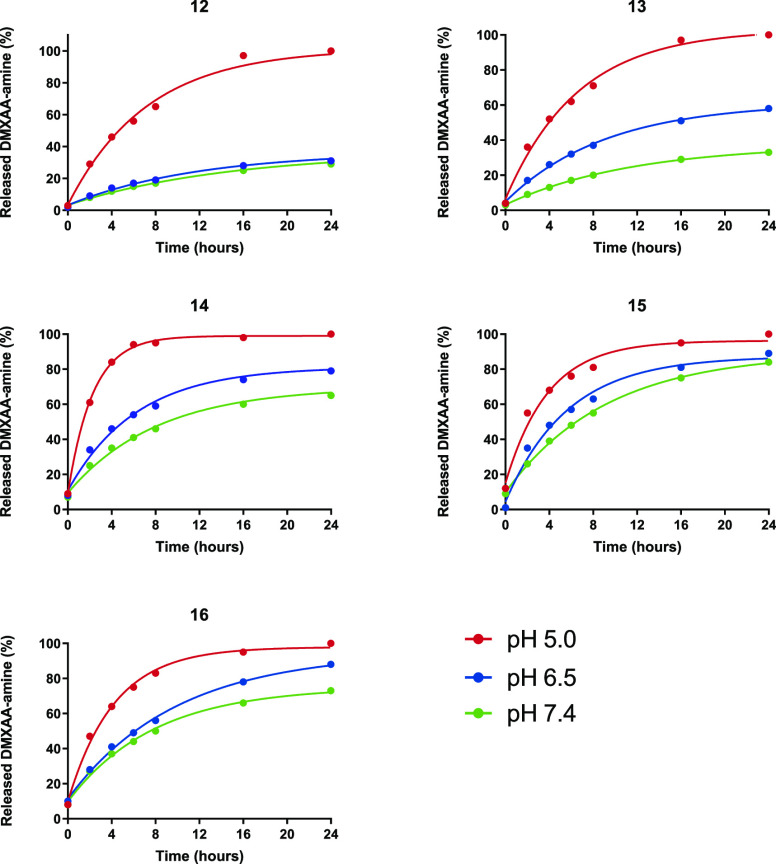
Release of DMXAA-amine **1** from CCPMs at 37 °C.

Figure 5 shows the release of native DMXAA-amine **1** as an API from CCPMs with incorporated trityl-DMXAA-amine constructs **12**–**16** at 37 °C in buffers of different pH values. All half-life values at the different pH values (Table 3) increased compared to the API–linker constructs, demonstrating a delaying effect of the CCPMs on the release of API. An exception to these observations was construct **12**, which showed faster release at pH 7.4 and 6.5 when entrapped in the CCPM. The general delaying effect of the other constructs can be explained by the lower water content, and thus H_3_O^+^ activity, in the hydrophobic and partly dehydrated core,^74^ which in turn leads to less protonation of the trityl-bound amine.

**Table 3:** *t*_1/2_ Values (in h) of DMXAA-Amine **1** Release from CCPMs at 37 °C

	pH 5.0 (h)	pH 6.5 (h)	pH 7.4 (h)
**12**	5.0 ± 1.4	ND	ND
**13**	4.6 ± 0.8	13.9 ± 2.4	ND
**14**	1.6 ± 0.1	5.1 ± 2.6	9.1 ± 3.4
**15**	2.7 ± 0.9	4.5 ± 1.9	6.5 ± 0.9
**16**	2.9 ± 0.7	6.2 ± 1.3	7.5 ± 1.4

**Figure 6:**
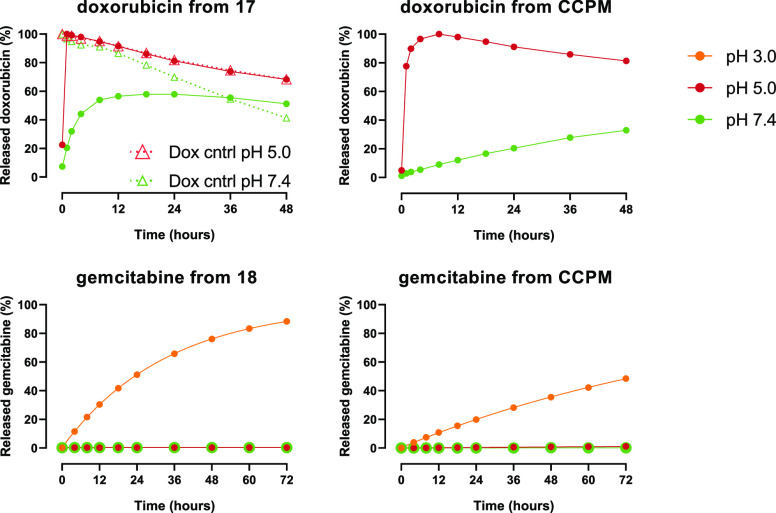
Release of gemcitabine and doxorubicin from trityl linker constructs **17** and **18**, both from the construct and from the core of CCPMs at different pH values at 37 °C.

**Scheme 3:**
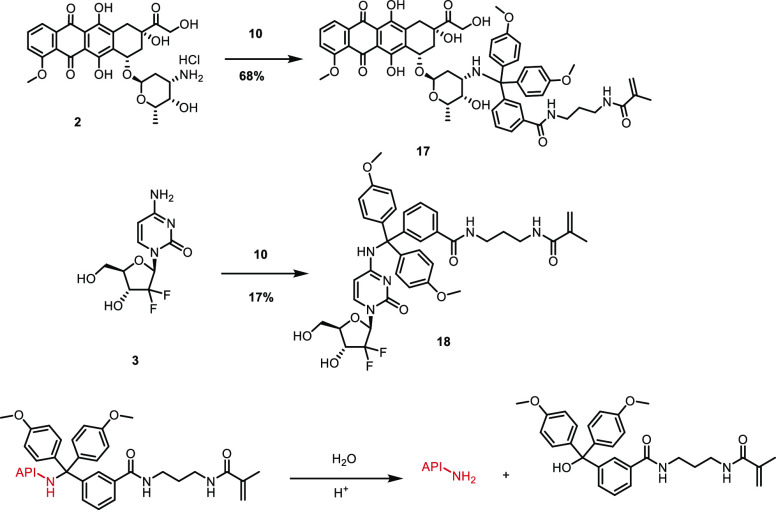
Synthesis Route of Amine-bearing APIs (Doxorubicin and Gemcitabine) with the Trityl Linker Having a Polymerizable Methacrylamide Moiety and Subsequent Release of Native API from the Construct

The (*para*)di-methoxy trityl linker in construct **12** is considered the most promising in view of the large difference between release at pH 5.0 and limited release of circa 30% within 24 h at pH 7.4 (Figure 6). This linker was therefore chosen for attachment to the two other model compounds, doxorubicin (**2**) and gemcitabine (**3**), to investigate the effect of the type of amine present on the API on the release kinetics (Scheme 3). Linker attachment to gemcitabine **3** was performed under similar conditions as described in Scheme 2. This resulted in the gemcitabine–linker construct **18**. Introduction of **10** to the primary amine functionality on the glucose ring of doxorubicin **2** did not require protection of the hydroxy functionalities as the amine is sufficiently nucleophilic and resulted directly in the doxorubicin–linker construct **17**. A detailed synthesis description is provided in S2.6 and S2.7. The release of doxorubicin and gemcitabine from the synthesized constructs was measured at different pH values following the same procedure as for DMXAA-amine.

Figure 6 shows that constructs **17** and **18** differed considerably in their release rates as compared to conjugate **12**. The gemcitabine–trityl construct **18** was much more stable at pH 7.4 and 5.0 (*t*_1/2_ value >72 h), whereas doxorubicin–trityl **17** showed a much faster release (*t*_1/2_ values of 44 and <1 h, respectively, Table 4). Figure 6 also shows that at pH 5.0, the doxorubicin–trityl construct **17** and the construct **17** in CCPMs rapidly released doxorubicin. However, upon further incubation, the concentration of doxorubicin decreased in time (also observed for the control of free doxorubicin in solution). The UHPLC analysis showed that no degradation products were formed and therefore the decrease in the doxorubicin concentration is likely caused by its dimerization followed by precipitation,^75^ which was also observed upon visual inspection of the HPLC vial. Therefore, the reported *t*_1/2_ values have to be considered as an estimation based on the first time points where decrease of control doxorubicin was still minimal. To get insight into the underlying mechanism of hydrolytic cleavage, the release of gemcitabine–trityl construct **18** at pH 3.0 was also measured.

**Table 4:** *t*_1/2_ Values (in h) for the Studied API–Trityl Constructs at 37 °C

	pH 3.0 (h)	pH 5.0 (h)	pH 7.4 (h)
DMXAA–trityl **12**	NM^d^	1.8 ± 0.1	ND
**12** entrapped in CCPM	NM^d^	5.0 ± 1.4	ND
dox–trityl **17**	NM^d^	<1	6^c^
**17** entrapped in CCPM	NM^d^	<1	44^c^
gem–trityl **18**	23.2 ± 0.5	ND	ND
**18** entrapped in CCPM	76.0 ± 3.0	ND	ND
LY207702^a^	NM^d^	3.71^b^	146

a LY207702 adapted from the study of Patel et al. as a reference.^71^

b Measured at pH = 5.4.

c Due to precipitation of doxorubicin, this value is an estimation.

d Not measured.

The half-lives of **12** and **17** at pH 5.0 and 37 °C are 1.8 and <1 h, respectively. The half-life of **18** under the same conditions could not be determined in this timeframe as the release was too slow, and control measurements of gemcitabine in the buffer gave no indication of degradation or precipitation.

Since in general the first step in the removal of a trityl protecting group is protonation of the amine functional group,^76^ it is hypothesized that easier protonation of the amine will lead to a faster cleavage of the trityl linker and formation of the native compound, as is shown by the mechanism depicted in Scheme 1.

The mechanism stated above is in agreement with the finding that the trityl linker doxorubicin conjugate **17** with the most basic nitrogen (pK_a_ 8.4)^77^ indeed released the API the fastest (*t*_1/2_ < 1 h at pH 5.0). The trityl linker gemcitabine conjugate **18** showed the slowest release (*t*_1/2_ = 23.3 h at pH 3.0) of the native compound, which corresponds to the weakly basic character of this nitrogen (pK_a_ 3.6, calculated).

A release was observed at pH 3.0 for gemcitabine derivatives, while no release was observed at pH 5.0 within 72 h despite the fact that still a small fraction (ca. 4%) of the amines is protonated based on the pK_a_. This could possibly be explained by the deviation of this calculated pK_a_ value from the actual value or protonation elsewhere of the cytosine moiety leading to a less effective removal of the trityl linker.

**Figure 7:**
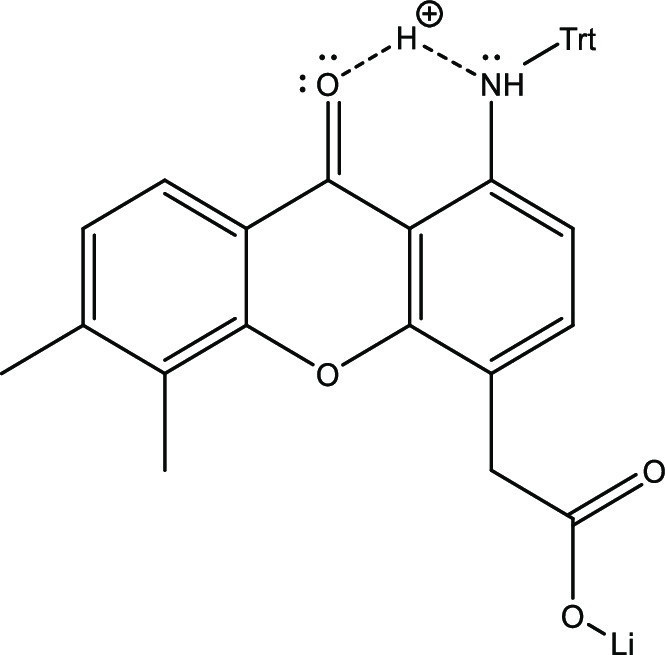
Facilitation of protonation of amine by adjacent carbonyl in DMXAA-amine.

In the DMXAA-amine-trityl linker construct **12**, despite the apparent weakly basic character of the nitrogen atom, a substantial release was observed at both pH 7.4 and 5. As shown in Figure 7, this high reactivity is explained by the previously described role of the carbonyl function adjacent to the amine, which facilitates hydrogen bonding and thus protonation of the trityl nitrogen.^78^

An important consequence of the above observations is that the pK_a_ of the involved nitrogen of the API can be used to predict the relative release rate under acidic conditions. Thus, an API with a more basic nitrogen (higher pK_a_) will be released faster than an API connected via a less basic nitrogen (lower pK_a_). However, as was shown for the release of DMXAA from construct **12**, it is also important to consider additional structural factors that can promote protonation and thereby release. The release kinetics of API–linker constructs **12**, **17**, and **18** from CCPMs were determined (Figure 6). Not unexpectedly, when entrapped in the hydrophobic core of these micelles, the release was slower than observed for the soluble conjugates in buffer. This finding strengthens the hypothesis that protonation is required for trityl release as in the hydrophobic core, the water activity is lower than that in buffer, which in turn impedes protonation of the conjugated amine.^79,80^

In conclusion, a novel class of linkers was developed based on the trityl group, which allows for the release of native APIs under slightly acidic conditions. Furthermore, the release profiles could be adjusted by varying the substituent pattern on the aromatic rings of the trityl moiety, achieving a *t*_1/2_ value between 1.6 and 5.0 h. Moreover, the nature of the amine conjugated to the trityl linker induced considerable variation of the release kinetics. The release kinetics varied from *t*_1/2_ = 5.0 h to no detectable release in 72 h. Using doxorubicin and gemcitabine as model APIs demonstrated that the release kinetics are dependent on both the conjugated system and the pK_a_ of the protected amine. The novel constructs were entrapped in CCPMs and maintained the desired acid-sensitive release profile of the API. Release from CCPMs was slower than release of API from the construct, which is most likely due to a lower water activity in the dehydrated hydrophobic core of the micelles.

These obtained insights on substituted trityl linkers can be of excellent use in the design of CCPMs with predictable release kinetics of entrapped APIs. Nevertheless, for any new API, with a specific mode of action, toxicity studies should be performed on the final CCPMs to investigate whether the chosen trityl linker is contributing to any observed toxicity.

Although we have shown that these bioconjugation principles can be applied to small-molecule APIs, we are confident that these principles can be extended to incorporation and release of peptides, siRNA, or other small biomolecules, which is currently under investigation.

## Supporting Information Available

Detailed synthesis of compounds **12–18**; protocol of API release measurement; and protocol of CCPM formulation (PDF)

## Abbreviations

API: active pharmaceutical ingredient
CCPM: core-cross-linked polymeric micelle
PEG: poly(ethylene glycol)
TME: tumor microenvironment
