# Supplementary material for: A New Class of Tunable Acid-Sensitive Linkers for Native Drug Release Based on the Trityl Protecting Group
Source: Bioconjug Chem. 2022 Aug 18;33(9):1707–15. doi: 10.1021/acs.bioconjchem.2c00310 (PMC9501768; doi:10.1021/acs.bioconjchem.2c00310)
Supplement: Supplementary file 1 — bc2c00310_si_001.pdf [file bc2c00310_si_001.pdf]

SUPPORTING INFO

A new class of tuneable acid-sensitive linkers for native drug release based on the trityl protecting group

Matt Timmers[a,b,*], Jimmy Weterings[a], Michiel van Geijn[a], Roel Bell[c], Peter E. Lenting[d], Cristianne J.F. Rijcken[a], Tina Vermonden[b], Wim E. Hennink[b], Rob M.J. Liskamp[a,d,e,*].

[a]*Cristal Therapeutics, Maastricht 6229 EV, the Netherlands*

[b]*Department of Pharmaceutics, Utrecht Institute for Pharmaceutical Sciences, Utrecht University, Utrecht 3584 CG, the Netherlands*

[c]*Symeres, Nijmegen 6546 BB, the Netherlands*

[d]*School of Chemistry, University of Glasgow, Glasgow G12 8QQ, United Kingdom*

[e]*Department of Biochemistry, Cardiovascular Research Institute Maastricht (CARIM), Maastricht University, Maastricht 6229 ER, the Netherlands*

**Corresponding authors*

Email: Robert.liskamp@glasgow.ac.uk

S1 General procedures s2
S2: synthesis of trityl-API constructs **12-16**, **17**, **18** s3
S2.1: Synthesis of **12** s3
S2.2**:** Synthesis of **13** s7
S2.3: Synthesis of **14** s12
S2.4:Synthesis of **15** s15
S2.5**:** Synthesis of **16** s18
S2.6: Synthesis of **17** s21
S2.7: Synthesis of **18** s23
S3: In vitro release protocol and stability control s24
S3.1 Release of API from API-linker construct s24
S3.2 Release of API from CCPM s26
S4: CCPM Formulation protocol s26

S1 General procedures

Reagents and starting materials were obtained from commercial suppliers unless otherwise noted. LCMS analysis was performed on an Agilent 1260; Bin. Pump: G1312B, degasser; autosampler, ColCom, DAD: Agilent G1315D, 220-320 nm, MSD: Agilent LC/MSD G6130B ESI, pos/neg 100-1000, ELSD Alltech 3300 gas flow 1.5 mL/min, gas temp: 40°C.

Method A, B: Eluent A: 0.1% formic acid in acetonitrile, Eluent B: 0.1% formic acid in water.

Method A: Column: Waters XSelect™ C18, 30x2.1mm, 3.5 μ, Temp: 35 °C, Flow: 1 mL/min, Gradient: t0 = 5% A, t1.6min = 98% A, t3min = 98% A, Posttime: 1.3 min.

Method B: Column: Waters XSelect™ CSH C18, 50x2.1mm, 3.5 μ, Temp: 40 °C, Flow: 0.8 mL/min, Gradient: t0 = 5% A, t4.5min = 98% A, t6min = 98% A, Posttime: 2 min.

Method C, D: Eluent A: acetonitrile, Eluent B: 10mM ammonium bicarbonate in water (pH=9.5).

Method C: Column: Waters XSelect™ CSH C18, 30x2.1mm, 3.5 μ, Temp: 25 °C, Flow: 1 mL/min, Gradient: t0 = 5% A, t1.6min = 98% A, t3min = 98% A, Posttime: 1.3 min.

Method D: Column: Waters XSelect™ CSH C18, 50x2.1mm, 3.5 μ, Temp: 25 °C, Flow: 0.8 mL/min, Gradient: t0 = 5% A, t3.5min = 98% A, t6min = 98% A, Posttime: 2 min.

Method E: Shimadzu, communication module (CBM-20A), autosampler (SIL-20HT), pump (LC-20AT), UV/Vis detector (SPD-20A), system controller (Labsolutions V5.54 SP). Eluent A: acetonitrile/water 5:95 with 0.1% TFA, Eluent B: acetonitrile/water 95:5 with 0.1% TFA. Column: Phenomenex Gemini C18 column, 110 Å, 5 µm, 250x4.6mm, Flow: 1 mL/min, Gradient: t0 = 100% A, t2min = 100% A, t32min = 0% A.

1H NMR spectra were recorded on a Bruker Avance-400 ultrashield NMR spectrometer, using CDCl3 or DMSO-d6 as solvent and are reported in ppm using TMS (0.00 ppm) as an internal standard.

S2: synthesis of trityl-API constructs **12-16**, **17**, **18**

*S2.1: Synthesis of **12***

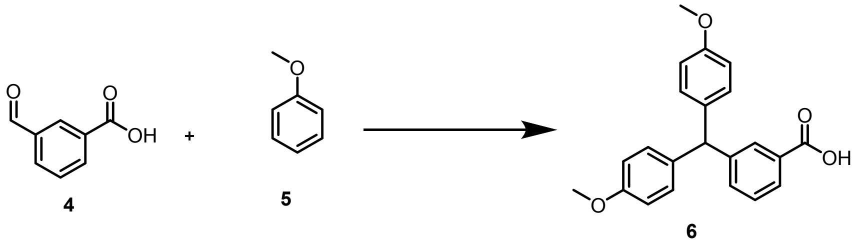

*3-(Bis(4-methoxyphenyl)methyl)benzoic acid (**6**).*

3-Formylbenzoic acid (**4**, 5.04 g, 33.6 mmol) and anisole (**5**, 8.51 mL, 78 mmol) were mixed with lukewarm acetic acid (107 mL) and stirred for 10 minutes. The mixture was warmed to 30 °C for 5 minutes in order to dissolve all of the 3-formylbenzoic acid and then cooled in an ice/water bath. Sulfuric acid (16.41 mL, 308 mmol) was added dropwise in 10 minutes and the cooling bath was subsequently removed and the mixture was allowed to warm to room temperature and stirred for 21 hours. Then, the mixture was poured into ice water (500 mL) and extracted with ethyl acetate (3 x 100 mL). The combined organic extracts were washed with water (2 x 50 mL) and brine (50 mL), dried over Na_2SO_4, filtered, and evaporated under reduced pressure. The residue was purified by repetitive flash column chromatography (silica, 5 to 50% ethyl acetate in n-heptane) to give 3-(bis(4-methoxyphenyl)methyl)benzoic acid (**6**, 4.99 g, 84% pure (LCMS), yield: 36%) as a colourless oil that partly solidified on standing. LCMS: 84%, RT = 2.14 min., (M+H)- = 347 (method A). ^{1}H NMR ($CDCl_3$): δ 7.98 – 7.92 (m, 1H), 7.89 (s, 1H), 7.42 – 7.32 (m, 2H), 7.00 (d, J = 8.7 Hz, 4H), 6.83 (d, J = 8.7 Hz, 4H), 5.51 (s, 1H), 3.79 (s, 6H).

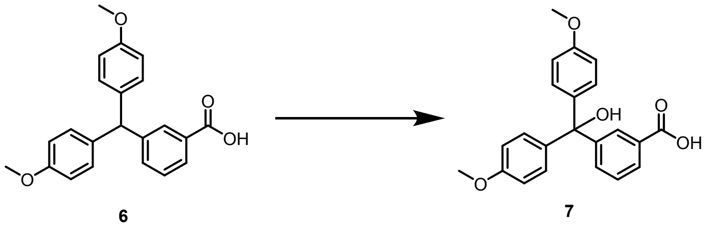

*3-(Hydroxybis(4-methoxyphenyl)methyl)benzoic acid **7***

Under a nitrogen atmosphere, manganese dioxide (5.6 g, 64.4 mmol) was added to a solution of 3-(bis(4-methoxyphenyl)methyl)benzoic acid (III, 4.50 g, 12.92 mmol) in lukewarm acetic acid (25 mL). The mixture was subsequently heated to 100 °C and stirred for 2 days. After cooling to room temperature, the reaction mixture was diluted with ethyl acetate and filtered through a layer of Celite. The filtrate was evaporated under reduced pressure and subsequently coevaporated with toluene. The residue was taken up in dichloromethane, coated on Isolute, and purified by flash column chromatography (silica, 0.1 to 3.5% methanol in dichloromethane) to give 3-(hydroxybis(4-methoxyphenyl)methyl)benzoic acid (1.1 g, 23%) as an off-white foam. LCMS: 97%, RT = 2.57 min., $(M+H)^-$ = 363 (method D). ^{1}H-NMR ($CDCl_3$): δ 8.08 (t, *J* = 1.8 Hz, 1H), 8.00 (dt, *J* = 7.7, 1.5 Hz, 1H), 7.57 (dt, *J* = 7.8, 1.5 Hz, 1H), 7.41 (t, *J* = 7.8 Hz, 1H), 7.20 – 7.13 (m, 4H), 6.87 – 6.81 (m, 4H), 3.80 (s, 6H).(Figure S1)

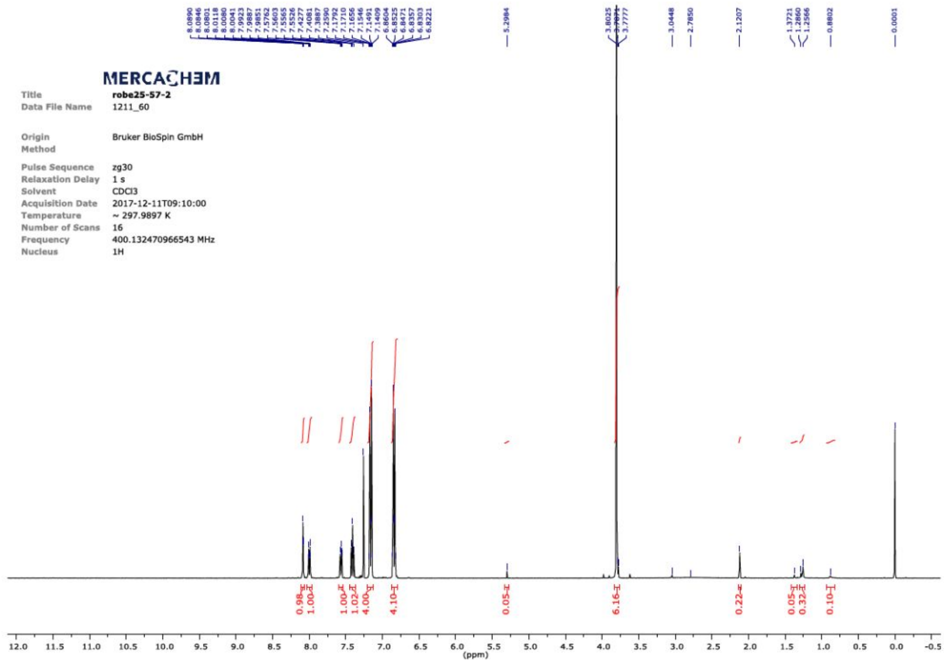

Figure S 1: NMR spectrum of 7

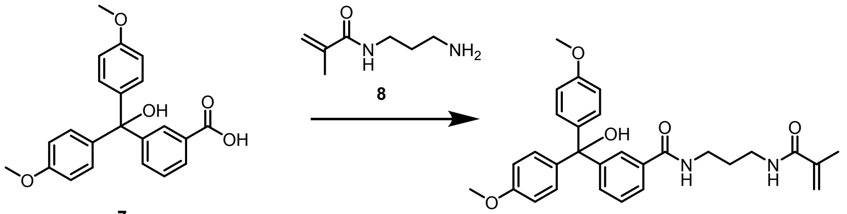

7

9

*3-(Hydroxybis(4-methoxyphenyl)methyl)-N-(3-methacrylamidopropyl)benzamide (**9**).*

Under a nitrogen atmosphere, *N*-(3-aminopropyl)methacrylamide hydrochloride (**8**, 1.030 g, 5.77 mmol) followed by *N*-(3-dimethylaminopropyl)-*N*′-ethylcarbodiimide hydrochloride (1.105 g, 5.77 mmol) were added to a solution of 3-(hydroxybis(4-methoxyphenyl)methyl)benzoic acid (**7**,1.91 g, 5.24 mmol) and *N*,*N*-diisopropylethylamine (2.014 mL, 11.53 mmol) in dichloromethane (40 mL). After stirring overnight, the conversion was 80% based on TLC. The mixture was washed with aqueous solution of citric acid (10% (w/w), 50 mL) and saturated aqueous $NaHCO_3$ solution (25 mL), dried over Na_2SO_4, and concentrated under reduced pressure. The residue was purified by flash column chromatography (silica, 60 to 100% ethyl acetate in *n*-heptane (containing 0.1-0.2% (v/v) triethylamine)) to give 3-(hydroxybis(4-methoxyphenyl)methyl)-*N*-(3-methacrylamidopropyl)benzamide (**9**, 1.82 g, 68%) as a white foam. LCMS: 99%, RT = 1.94 min., $(M+H)^-$ = 487 (method C). ^{1}H-NMR ($CDCl_3$): δ 7.93 (t, *J* = 1.8 Hz, 1H), 7.75 (dt, *J* = 7.4, 1.7 Hz, 1H), 7.40 – 7.30 (m, 2H), 7.20 – 7.12 (m, 4H), 7.05 – 6.96 (m, 1H), 6.87 – 6.80 (m, 4H), 6.68 – 6.59 (br s, 1H), 5.77 (s, 1H), 5.35 (q, *J* = 1.4 Hz, 1H), 3.80 (s, 6H), 3.47 (q, *J* = 6.2 Hz, 2H), 3.39 (q, *J* = 6.3 Hz, 2H), 2.97 (s, 1H), 1.98 (s, 3H), 1.79 – 1.70 (m, 2H).(Figure S2)

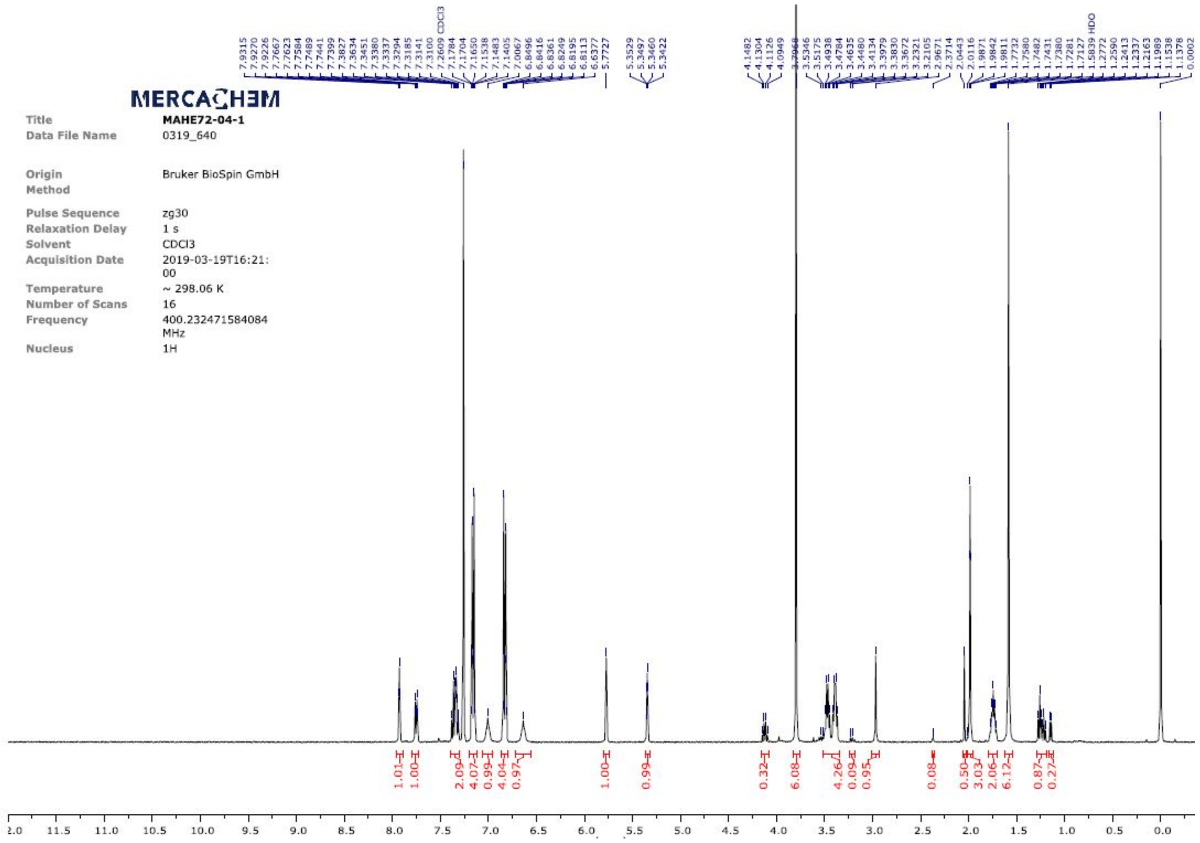

Figure S 2: NMR spectrum of 8

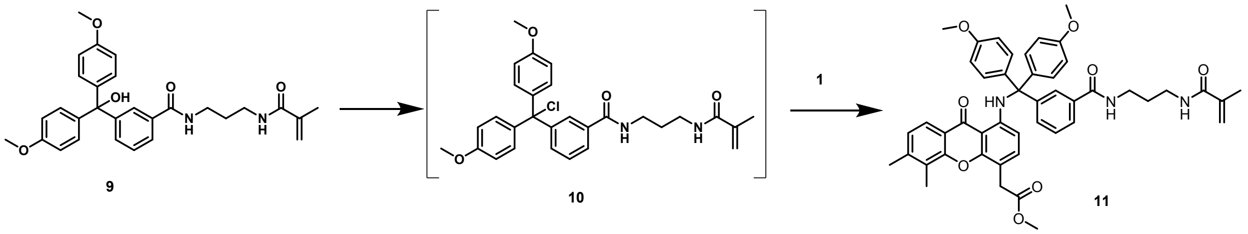

*3-(Chlorobis(4-methoxyphenyl)methyl)-N-(3-methacrylamidopropyl)benzamide (**10**).*

Under a nitrogen atmosphere, acetyl chloride (5.0 mL, 70.3 mmol) was added to 3-(hydroxybis(4-methoxyphenyl)methyl)-*N*-(3-methacrylamidopropyl)benzamide (**9**, 1.01 g, 1.96 mmol). After stirring for 15 minutes, the red mixture was concentrated *in vacuo* and pressurized with argon. The residue was decanted from *n*-heptane (2 x 25 mL), coevaporated from *n*-heptane (25 mL) and placed under argon atmosphere to give 3-(chlorobis(4-methoxyphenyl)methyl)-*N*-(3-methacrylamidopropyl)-benzamide (**10**, 1.96 mmol) which was used as such.

*Methyl 2-(1-(((3-((3-methacrylamidopropyl)carbamoyl)phenyl)bis(4-methoxyphenyl)methyl)amino)-5,6-dimethyl-9-oxo-9H-xanthen-4-yl)acetate **11**.*

To 3-(chlorobis(4-methoxyphenyl)methyl)-*N*-(3-methacrylamidopropyl)benzamide (**10**, 1.96 mmol), methyl 2-(1-amino-5,6-dimethyl-9-oxo-9*H*-xanthen-4-yl)acetate (619 mg, 1.99 mmol) and pyridine (10.0 mL, 124 mmol) were added. After stirring for 15 minutes the bright yellow solution was extracted between dichloromethane (50 mL) and aqueous saturated $NaHCO_3$ solution (25 mL). The aqueous layer was extracted with dichloromethane (20 mL) and the combined organic layers were dried over Na_2SO_4, filtered and concentrated *in vacuo*. The residue was purified by repetitive basic preparative MPLC (Waters XSelect CSH C18 (145x25 mm, 10µ), linear gradient: t=0 min 50% B; t=4 min 50% B; t=16 min 100% B; t=21 min 100% B; detection: ELSD) and combined product fractions were lyophilized to

give methyl 2-(1-(((3-((3-methacrylamidopropyl)carbamoyl)phenyl)bis(4-methoxyphenyl)methyl)amino)-5,6-dimethyl-9-oxo-9*H*-xanthen-4-yl)acetate (**11**, 1.00 g, 65% yield) as a yellow fluffy solid. LCMS: 100%, RT = 2.38 min., $[M-H]^-$ = 780 (method C). ^1H NMR (400 MHz, $CDCl_3$) δ 11.34 (s, 1H), 8.01 – 7.91 (m, 2H), 7.72 (d, *J* = 7.6 Hz, 1H), 7.50 (d, *J* = 8.0 Hz, 1H), 7.37 (t, *J* = 7.8 Hz, 1H), 7.33 – 7.27 (m, 4H), 7.14 (d, *J* = 8.3 Hz, 1H), 6.96 (dd, *J* = 17.9, 7.6 Hz, 2H), 6.85 – 6.78 (m, 4H), 6.74 – 6.61 (m, 1H), 5.86 (d, *J* = 8.7 Hz, 1H), 5.75 (s, 1H), 5.31 (t, *J* = 1.5 Hz, 1H), 3.78 (s, 6H), 3.71 – 3.67 (m, 5H), 3.46 (q, *J* = 6.2 Hz, 2H), 3.34 (q, *J* = 6.2 Hz, 2H), 2.42 (s, 3H), 2.38 (s, 3H), 1.96 (s, 3H), 1.77 – 1.68 (m, 2H). (Figure S3)

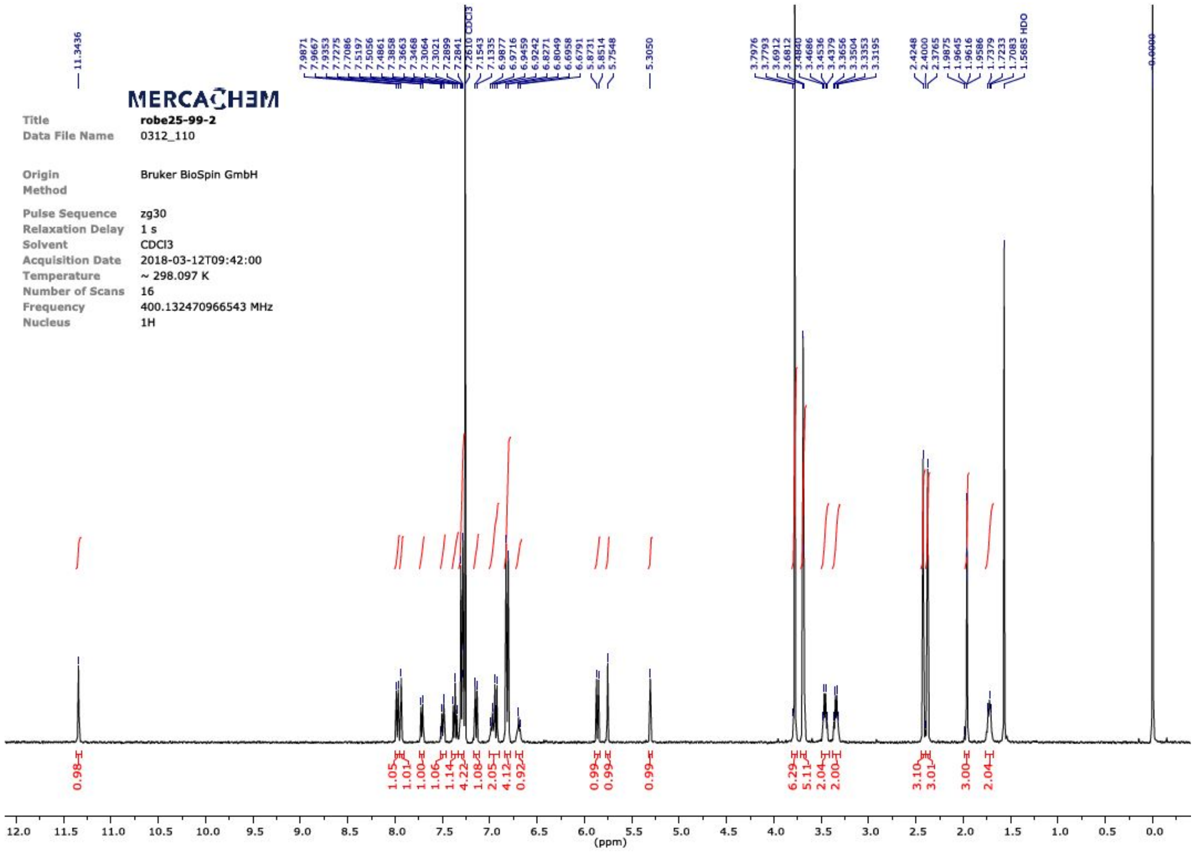

Figure S 3: NMR spectrum of 11

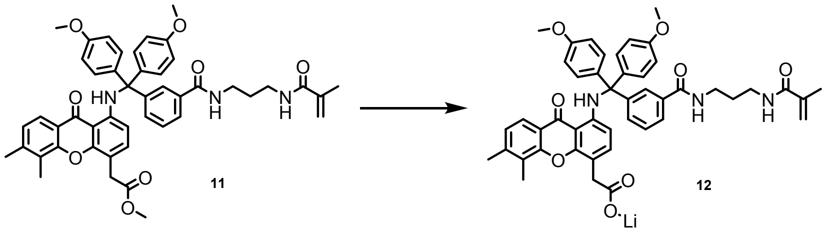

*Lithium 2-(1-(((3-((3-methacrylamidopropyl)carbamoyl)phenyl)bis(4-methoxyphenyl)methyl)amino)-5,6-dimethyl-9-oxo-9H-xanthen-4-yl)acetate (**12**).*

Under a nitrogen atmosphere, a solution of lithium hydroxide monohydrate (56 mg, 1.33 mmol) in water (6.0 mL) was added to a solution of methyl 2-(1-(((3-((3-methacrylamido-propyl)carbamoyl)phenyl)bis(4-methoxyphenyl)methyl)amino)-5,6-dimethyl-9-oxo-9*H*-xanthen-4-yl)acetate (**11**, 0.98 g, 1.25 mmol) in tetrahydrofuran (15 mL) and the slightly turbid mixture was stirred at room temperature overnight. The now clear yellow mixture was concentrated *in vacuo* and the aqueous residue was diluted with water (2.0 mL), and purified by basic preparative MPLC (Waters

XSelect CSH C18 (145x25 mm, 10µm), linear gradient: t=0 min 5% B; t=3 min 50% B; t=19 min 60% B; t=21 min 100% B; detection: ELSD). Combined products fractions were lyophilized to afford lithium 2-(1-(((3-((3-methacrylamidopropyl)carbamoyl)phenyl)bis(4-methoxyphenyl)methyl)amino)-5,6-dimethyl-9-oxo-9*H*-xanthen-4-yl)acetate (**12**, 0.84 g, 79%) as a fluffy yellow solid. LCMS: 91%, RT = 3.06 min.

Expected mass: 773.8, found: $[M\text{-}Li]^-$ = 766.

^{1}H NMR (400 MHz, DMSO-d_6) δ 11.19 (s, 1H), 8.48 (t, *J* = 5.8 Hz, 1H), 7.96 – 7.82 (m, 3H), 7.72 (d, *J* = 7.3 Hz, 1H), 7.51 – 7.39 (m, 2H), 7.27 – 7.17 (m, 5H), 7.11 – 7.03 (m, 1H), 6.96 – 6.88 (m, 4H), 5.78 (d, *J* = 8.7 Hz, 1H), 5.62 (s, 1H), 5.29 (s, 1H), 3.74 (s, 6H), 3.60 (s, 2H), 3.21 (q, *J* = 6.7 Hz, 2H), 3.12 (q, *J* = 6.6 Hz, 2H), 2.40 (s, 3H), 2.33 (s, 3H), 1.83 (s, 3H), 1.64 (p, *J* = 6.8 Hz, 2H).

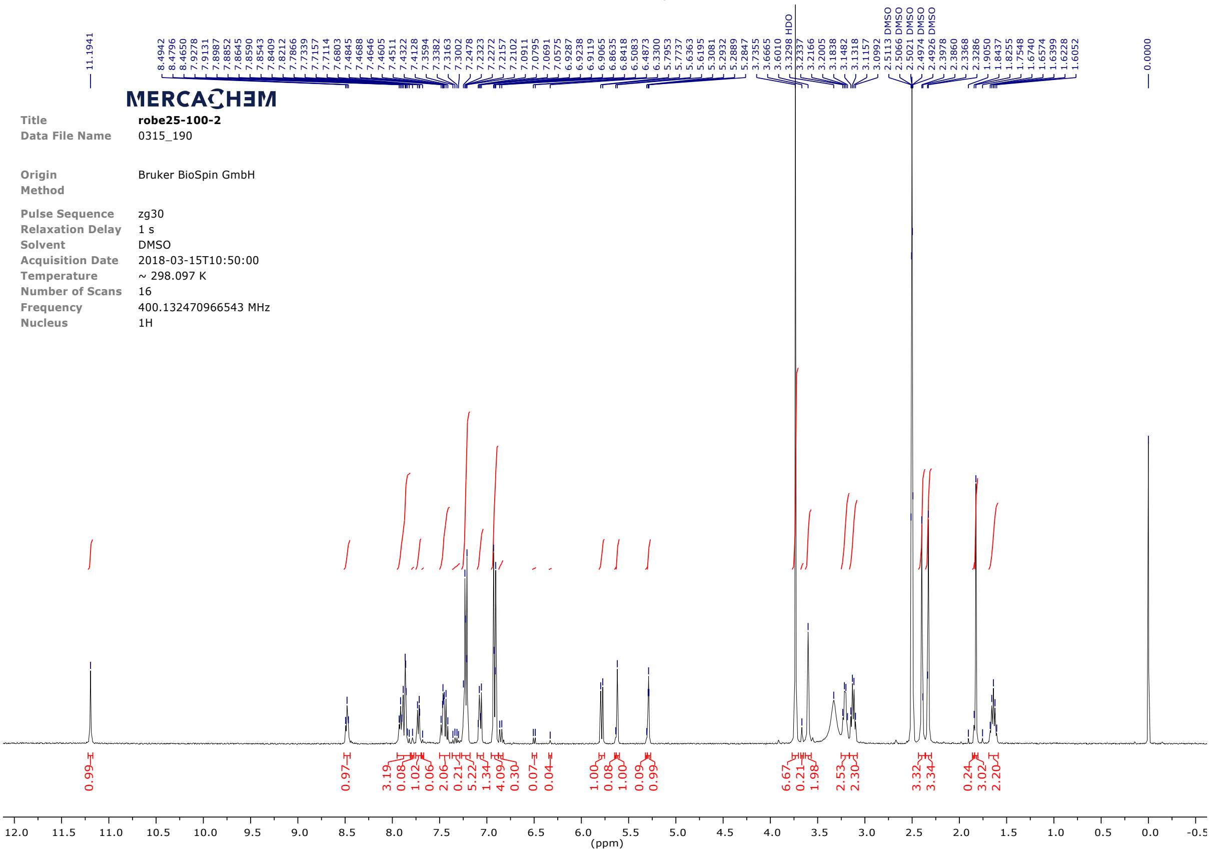

*Contains ~6% (w/w) compound **9** and ~4% (w/w) compound **1***

S2.2: Synthesis of 13

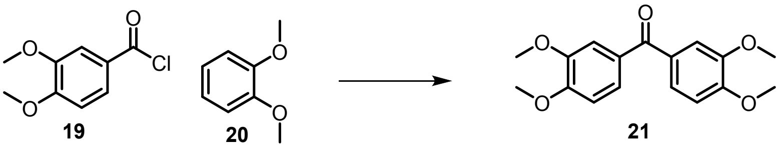

*Bis(3,4-dimethoxyphenyl)methanone (**21**).*

Under a nitrogen atmosphere, 1,2-dimethoxybenzene (**20**, 3.41 ml, 26.8 mmol) and 3,4-dimethoxybenzoyl chloride (**19**, 5.37 g, 26.8 mmol) were dissolved in dry dichloromethane (60 mL) and aluminium chloride (4.82 g, 36.1 mmol) was added in small portions. The obtained red mixture was divided in four batches and heated in closed flasks at 60 °C for 4 hours. The mixtures were cooled down, combined and poured into cold aqueous HCl (1M, 50 mL), and diluted with dichloromethane (25 mL). The layers were separated and the aqueous phase was extracted with dichloromethane (25 mL). The combined organic layers were washed with water (25 mL), dried over Na_2SO_4, filtered, and

evaporated under reduced pressure. The residue was crystallized from ethanol overnight. Formed crystals were collected, washed twice with ethanol and twice with diisopropyl ether, and dried by air current on the filter to give bis(3,4-dimethoxyphenyl)methanone (**21**, 5.52 g, 68% yield) as a red/brown solid. ^{1}H NMR(400 MHz, $CDCl_3$) δ 7.44 (d, *J* = 2.0 Hz, 2H), 7.39 (dd, *J* = 8.3, 2.0 Hz, 2H), 6.91 (d, *J* = 8.3 Hz, 2H), 3.97 (s, 6H), 3.95 (s, 6H). (Figure S4)

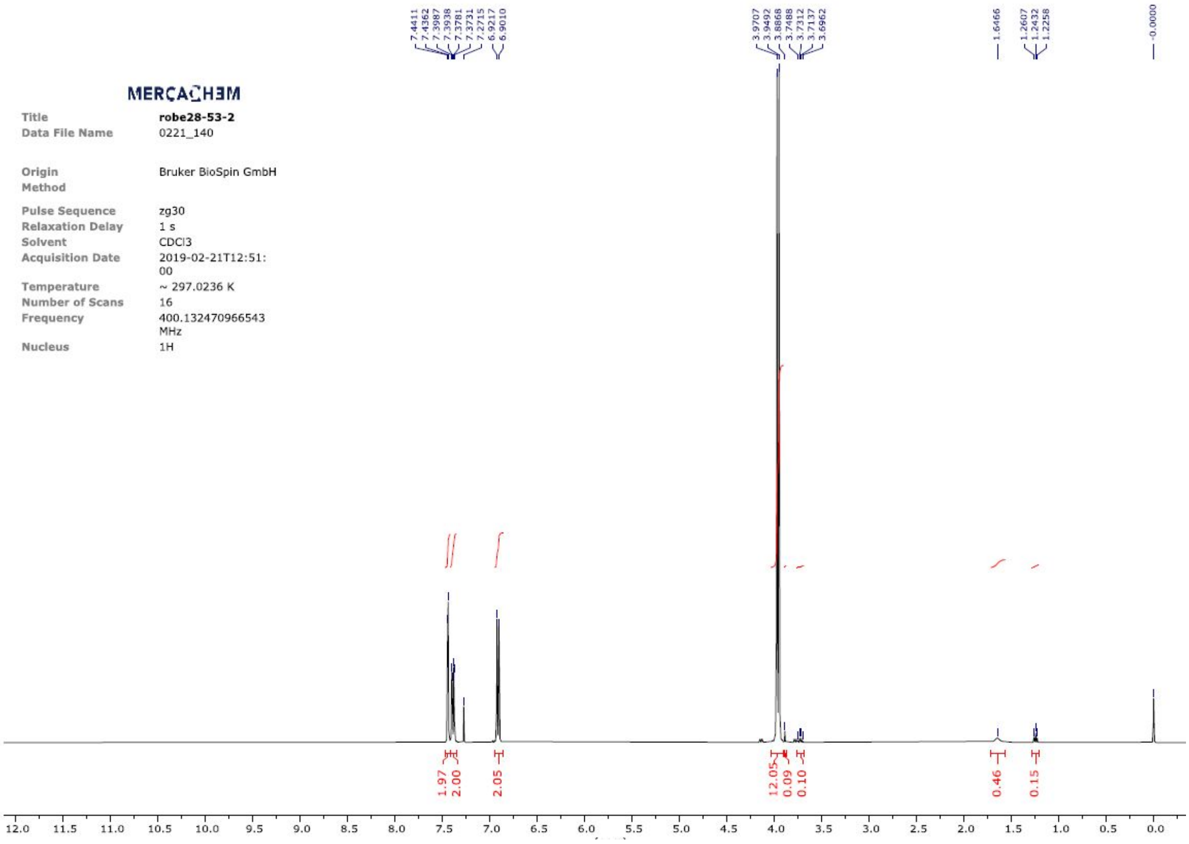

Figure S 4: NMR spectrum of 21

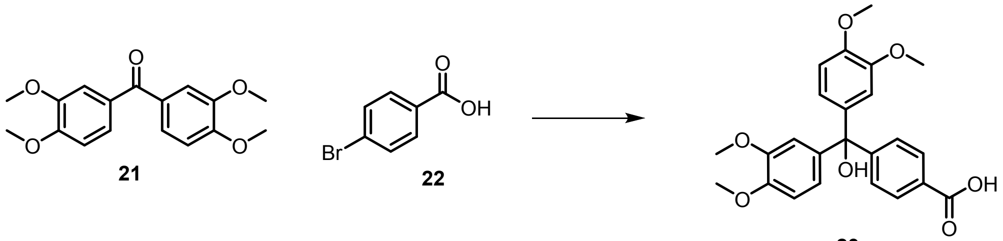

*4-(Bis(3,4-dimethoxyphenyl)(hydroxy)methyl)benzoic acid (**23**).*

Under a nitrogen atmosphere, 4-bromobenzoic acid (3.0 g, 14.9 mmol) was dissolved in tetrahydrofuran (dry, 30 mL) and cooled to -15 °C. A solution of di-*n*-butylmagnesium (1.0 M in *n*-heptane, 7.81 mL, 7.81 mmol) was added via syringe over 15 minutes while the temperature of the milky suspension was kept below -10 °C. After 10 minutes, a solution of *n*-butyllithium (2.5 M in hexanes, 6.27 mL, 15.67 mmol) was added via syringe over 15 minutes. After another 15 minutes, a solution of bis(3,4-dimethoxyphenyl)methanone (**21**, 2.256 g, 7.46 mmol) in tetrahydrofuran (dry, 50 mL) was added via a dropping funnel in 20 minutes. Next, the light-yellow suspension was allowed to warm to room temperature overnight. The mixture was quenched with aqueous NaOH (1 M, 75 mL) and extracted with diethyl ether (2 x 75 mL). The aqueous layer was acidified with aqueous HCl (2 M, 75 mL) and the formed suspension was extracted with ethyl acetate (2 x 200 mL). The combined

organic layers were dried (Na_2SO_4), filtered, and evaporated under reduced pressure to give 4-(bis(3,4-dimethoxyphenyl)(hydroxy)methyl)benzoic acid (**23**, 4.38 g, 87% yield) as a yellow solid which was used as such. LCMS: 63%, RT = 1.51 min., $[M-H]^-$ = 423 (method C). ^1H NMR (400 MHz, DMSO-d_6) δ 12.94 (br s, 1H), 7.96 (d, *J* = 7.1 Hz, 2H), 7.88 (d, *J* = 8.4 Hz, 2H), 6.95 (d, *J* = 2.0 Hz, 2H), 6.84 (d, *J* = 8.5 Hz, 2H), 6.50 (dd, *J* = 8.4, 2.1 Hz, 2H), 3.73 (s, 6H), 3.63 (s, 6H).

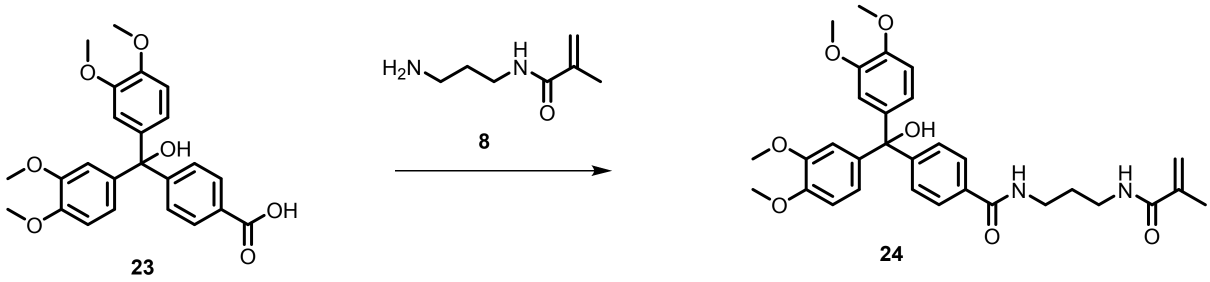

*4-(Bis(3,4-dimethoxyphenyl)(hydroxy)methyl)-N-(3-methacrylamidopropyl)benzamide (**24**).*
Under a nitrogen atmosphere, *N*,*N*-diisopropylethylamine (4.54 mL, 26.0 mmol) was added to a suspension of 4-(bis(3,4-dimethoxyphenyl)(hydroxy)methyl)benzoic acid (**23**, 4.38 g, 6.50 mmol) in dichloromethane (30 mL). *N*-(3-aminopropyl)methacrylamide hydrochloride (**7**, 2.32 g, 13.00 mmol) and *N*-(3-dimethylaminopropyl)-*N*′-ethylcarbodiimide hydrochloride (2.99 g, 15.60 mmol) were added and the mixture was stirred at room temperature overnight to yield a clear solution. More *N*,*N*-diisopropylethylamine (1.14 mL, 6.50 mmol), *N*-(3-aminopropyl)methacrylamide hydrochloride (581 mg, 3.25 mmol), and *N*-(3-dimethylaminopropyl)-*N*′-ethylcarbodiimide hydrochloride (623 mg, 3.25 mmol) were added and the mixture was stirred at room temperature for another 2 hours. The mixture was washed with aqueous citric acid (10% (w/w), 450 mL), aqueous saturated $NaHCO_3$ (500 mL), and brine (500 mL). All aqueous layers were combined and extracted with dichloromethane (50 mL) and the combined organic layers were dried over Na_2SO_4, filtered, and concentrated *in vacuo*. The residue was purified by flash column chromatography (silica, 60 to 100% ethyl acetate in *n*-heptane (containing 0.1% (v/v) triethylamine), detection: ELSD) to give 4-(bis(3,4-dimethoxyphenyl)(hydroxy)methyl)-*N*-(3-methacrylamidopropyl)benzamide (1.77 g, 50% yield). LCMS: 97%, RT = 1.79 min., $[M-H]^-$ = 547 (method C). ^1H NMR (400 MHz, $CDCl_3$) δ 7.81 (dd, *J* = 8.5, 1.9 Hz, 2H), 7.40 (d, *J* = 8.4 Hz, 2H), 7.21 (t, *J* = 6.3 Hz, 1H), 6.90 (d, *J* = 2.2 Hz, 2H), 6.77 (d, *J* = 8.6 Hz, 2H), 6.65 (dd, *J* = 8.4, 2.2 Hz, 2H), 6.56 – 6.47 (m, 1H), 5.78 (s, 1H), 5.36 (s, 1H), 3.88 (s, 6H), 3.77 (s, 6H), 3.53 – 3.38 (m, 4H), 2.80 (s, 1H), 2.00 (d, *J* = 1.3 Hz, 3H), 1.79 – 1.71 (m, 2H).(Figure S5)

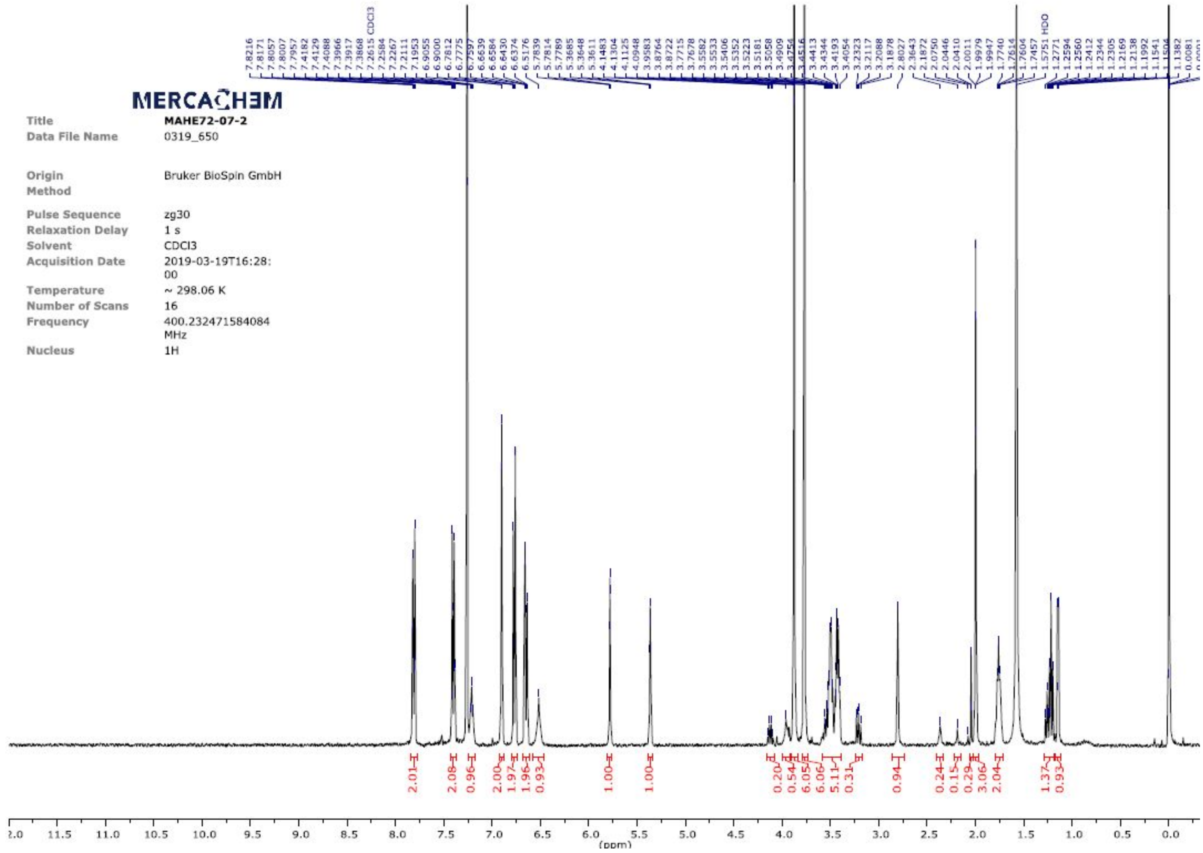

Figure S 5: NMR spectrum of 24

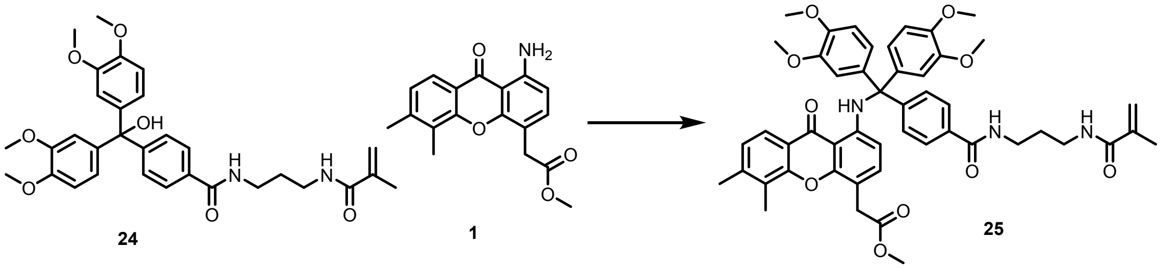

*Methyl2-(1-((bis(3,4-dimethoxyphenyl)(4-((3-methacrylamidopropyl)carbamoyl)phenyl)methyl)amino)-5,6-dimethyl-9-oxo-9H-xanthen-4-yl)acetate (**25**).*

Under an argon atmosphere, acetyl chloride (1.0 mL, 14.06 mmol) was added to 4-(bis(3,4-dimethoxyphenyl)(hydroxy)methyl)-*N*-(3-methacrylamidopropyl)benzamide (**24**, 253 mg, 0.46 mmol). After stirring for 10 minutes, the dark purple reaction mixture was concentrated *in vacuo*, coevaporated from *n*-heptane (2 x 10 mL) and pressurized with argon. Methyl 2-(1-amino-5,6-dimethyl-9-oxo-9*H*-xanthen-4-yl)acetate (**1**, 144 mg, 0.46 mmol) and pyridine (2.0 mL, 24.73 mmol) were added and the clear yellow solution was stirred for 10 minutes, and then partitioned between dichloromethane (20 mL) and saturated aqueous $NaHCO_3$ (10 mL). The organic layer was passed through a hydrophobic frit and evaporated under reduced pressure. The residue was purified by column chromatography (alumina (neutral), ethyl acetate (containing 1% (v/v) triethylamine)) to give methyl 2-(1-((bis(3,4-dimethoxyphenyl)(4-((3-methacrylamidopropyl)carbamoyl)phenyl)methyl)-amino)-5,6-dimethyl-9-oxo-9*H*-xanthen-4-yl)acetate (232 mg, 60% yield) as a yellow foam. LCMS: 93%, RT = 2.22 min., $[M-H]^-$ = 840 (method C). ^1H NMR (400 MHz, $CDCl_3$) δ 11.34 (s, 1H), 7.99 (d, *J* =

8.1 Hz, 1H), 7.80 (d, *J* = 8.5 Hz, 2H), 7.51 (d, *J* = 8.5 Hz, 2H), 7.31 – 7.23 (m, 1H), 7.14 (d, *J* = 8.2 Hz, 1H), 6.99 – 6.88 (m, 5H), 6.82 – 6.74 (m, 2H), 6.71 (t, *J* = 6.3 Hz, 1H), 5.91 (d, *J* = 8.7 Hz, 1H), 5.78 (s, 1H), 5.34 (s, 1H), 3.86 (s, 6H), 3.72 – 3.65 (m, 11H), 3.52 – 3.36 (m, 4H), 2.42 (s, 3H), 2.39 (s, 3H), 1.98 (s, 3H), 1.73 (p, J = 7.0 Hz, 2H).(Figure S6)

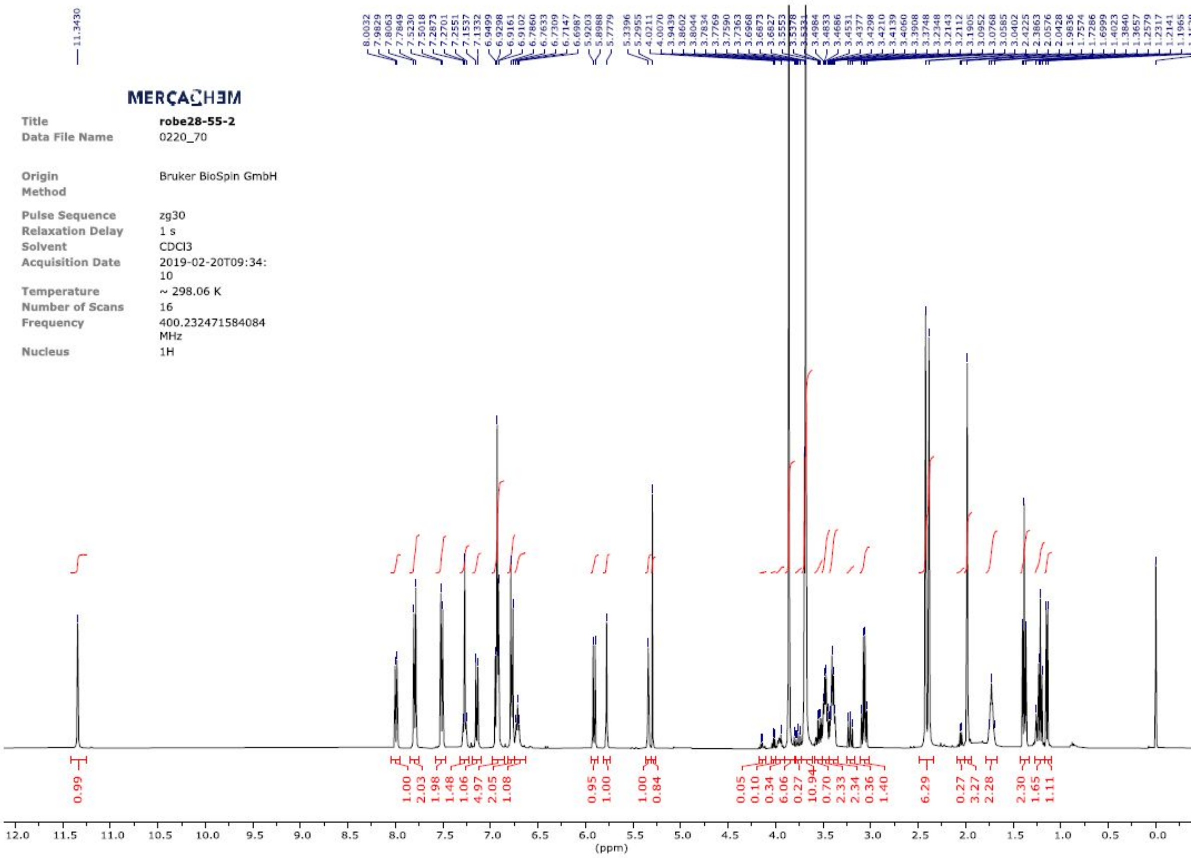

Figure S 6: NMR spectrum of 25

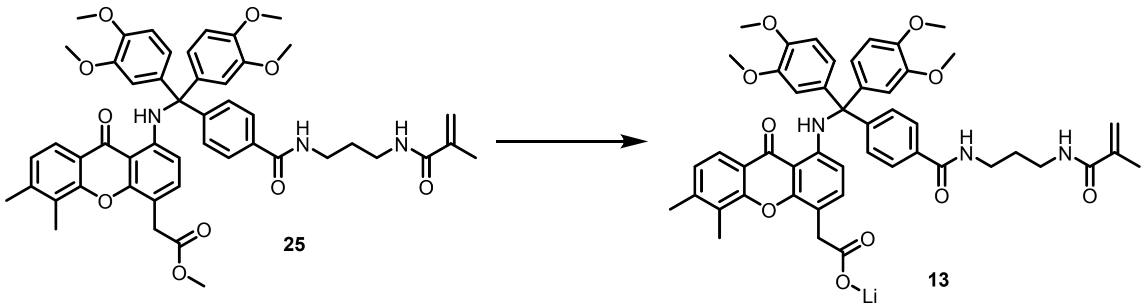

*Lithium2-(1-((bis(3,4-dimethoxyphenyl)(4-((3-methacrylamidopropyl)carbamoyl)phenyl)methyl)-amino)-5,6-dimethyl-9-oxo-9H-xanthen-4-yl)acetate (**13**).*

Under a nitrogen atmosphere, a solution of lithium hydroxide monohydrate (23.6 mg, 0.562 mmol) in water (1.6 mL) was added to a solution of methyl 2-(1-((bis(3,4-dimethoxyphenyl)(4-((3-methacrylamidopropyl)carbamoyl)phenyl)methyl)amino)-5,6-dimethyl-9-oxo-9*H*-xanthen-4-yl)acetate (**25**, 232 mg, 0.276 mmol) in tetrahydrofuran (4.0 mL) and the mixture was stirred at room temperature for 4 hours. The mixture was concentrated *in vacuo*, and the aqueous residue was diluted with water (1.0 mL) and acetonitrile (2.0 mL). The turbid mixture was filtered over a 45 µm nylon filter and purified by basic preparative MPLC (Waters XSelect CSH C18 (145x25 mm, 10µ), linear gradient: t=0 min 5% B; t=1 min 5% B; t=17 min 50% B; t=23 min 100% B; detection: ELSD). Combined product

fractions were lyophilized to give lithium 2-(1-((bis(3,4-dimethoxyphenyl)(4-((3-methacryl-amidopropyl)carbamoyl)phenyl)methyl)amino)-5,6-dimethyl-9-oxo-9*H*-xanthen-4-yl)acetate (**13**, 166 mg, 72%) as a yellow fluffy solid. LCMS: 97%, RT = 2.83 min.

Expected mass: 833.9, found $[M-Li]^-$ = 826 (method D).

^{1}H NMR (400 MHz, DMSO-d_6) δ 11.22 (s, 1H), 8.46 (t, *J* = 5.8 Hz, 1H), 7.93 (t, *J* = 5.8 Hz, 1H), 7.90 (d, *J* = 8.1 Hz, 1H), 7.78 (d, *J* = 8.3 Hz, 2H), 7.45 (d, *J* = 8.4 Hz, 2H), 7.26 (d, *J* = 8.2 Hz, 1H), 7.11 (d, *J* = 8.7 Hz, 1H), 6.94 (d, *J* = 8.3 Hz, 2H), 6.91 – 6.83 (m, 4H), 5.87 (d, *J* = 8.6 Hz, 1H), 5.64 (s, 1H), 5.33 – 5.28 (m, 1H), 3.74 (s, 6H), 3.62 (s, 2H), 3.57 (s, 6H), 3.25 (q, *J* = 6.6 Hz, 2H), 3.15 (q, *J* = 6.7 Hz, 2H), 2.41 (s, 3H), 2.35 (s, 3H), 1.84 (s, 3H), 1.66 (p, *J* = 6.9 Hz, 2H).

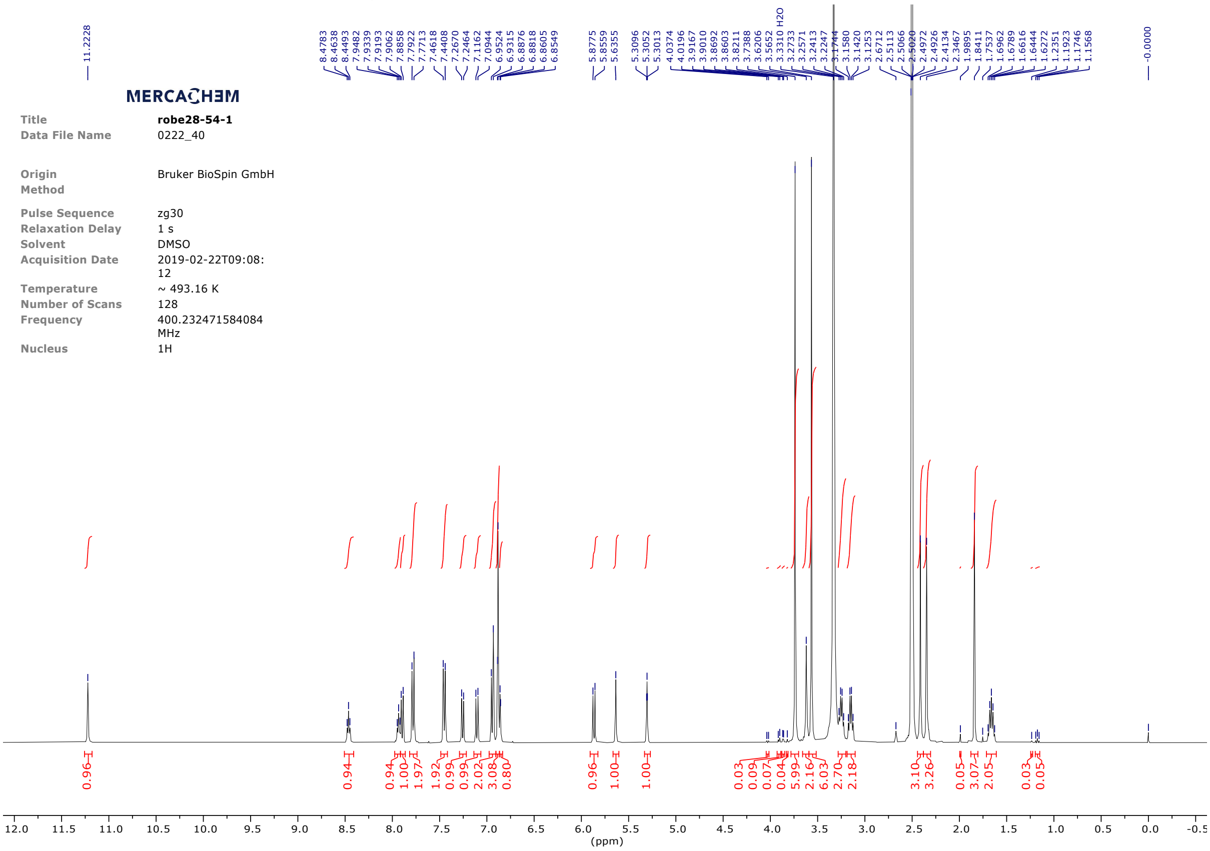

S2.3: Synthesis of 14

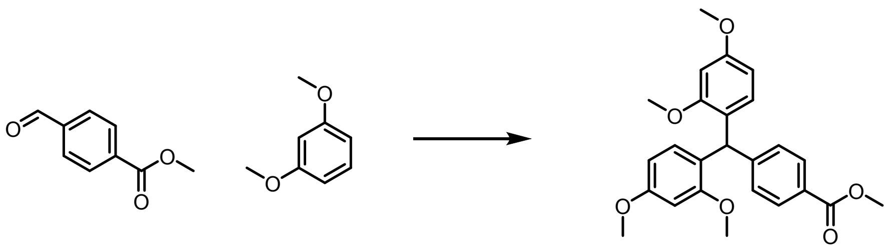

26

*Methyl 4-(bis(2,4-dimethyoxyphenyl)methyl)benzoate(**26**).*

A solution of methyl 4-formyl benzoate (2.47 g, 15.0 mmol) and 1,3-dimethoxybenzene (4.50 mL, 34.5 mmol) in ethyl acetate (48 mL) was cooled to 0 °C and concentrated sulfuric acid (7.4 mL) was added dropwise over 10 minutes. The mixture was stirred at room temperature overnight, washed with water (2 x 25 mL) and brine (25 mL) and dried over $MgSO_4$. After filtration, the filtrate was evaporated

under reduced pressure and the residue was purified by flash column chromatography (silica, 0 to 50% ethyl acetate in petroleum ether (boiling range 40-60 °C)) to give methyl 4-(bis(2,4-dimethyoxyphenyl)methyl)benzoate (**26**, 1.43 g, 23%) as a white solid. ^{1}H NMR (400 MHz, $CDCl_3$): δ 7.89 (d, *J* = 8.4 Hz, 2H), 7.10 (d, *J* = 8.1 Hz, 2H), 6.65 (dd, *J* = 8.4, 0.6 Hz, 2H), 6.46 (d, *J* = 2.5 Hz, 2H), 6.36 (dd, *J* = 8.4, 2.5 Hz, 2H), 6.03 (s, 1H), 3.88 (s, 3H), 3.78 (s, 6H), 3.66 (s, 6H).

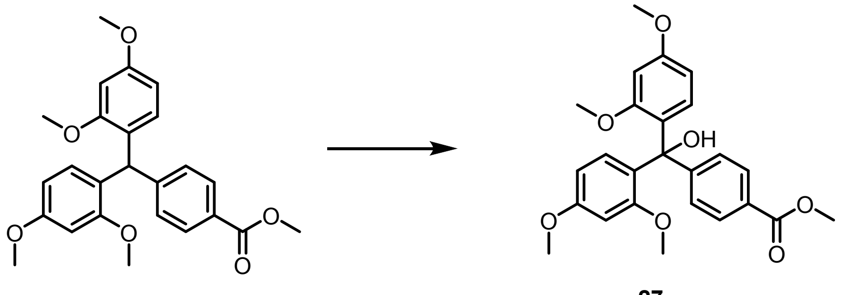

26 **27**

*Methyl 4-(bis(2,4-dimethoxyphenyl)(hydroxy)methyl)benzoate(**27**).*

A mixture of methyl 4-(bis(2,4-dimethyoxyphenyl)methyl)benzoate (**26**, 604 mg, 1.43 mmol) and manganese (IV) dioxide (1.87 g, 21.5 mmol) in toluene (10 mL) was stirred under reflux at 110 °C for 42 hours. The reaction mixture was cooled down, diluted with ethyl acetate (40 mL) and filtered through a pad of Celite. The filtrate was concentrated under reduced pressure and the residue was purified by flash column chromatography (silica, 0 to 50% ethyl acetate in petroleum ether (boiling range 40-60 °C), containing 0.1% (v/v) triethylamine) to give methyl 4-(bis(2,4-dimethoxyphenyl)(hydroxy)methyl)benzoate (63.3 mg, 10%) as an off-white foam. ^{1}H NMR (400 MHz, $CDCl_3$): δ 7.91 (d, *J* = 8.3 Hz, 2H), 7.33 (d, *J* = 8.2 Hz, 2H), 6.89 (d, *J* = 8.6 Hz, 2H), 6.49 (d, *J* = 2.5 Hz, 2H), 6.40 (dd, *J* = 8.7, 2.5 Hz, 2H), 5.12 (s, 1H), 3.90 (s, 3H), 3.80 (s, 6H), 3.51 (s, 6H).

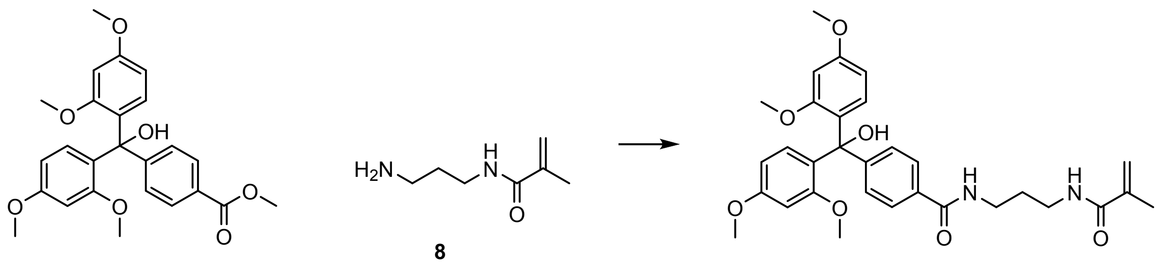

27 **8** **28**

*4-(Bis(2,4-dimethoxyphenyl)(hydroxy)methyl)-N-(3-methacrylamidopropyl)benzamide (**28**).*

A solution of NaOH (14.3 mg, 0.357 mmol) in water (2 mL) was added to a solution of methyl 4-(bis(2,4-dimethoxyphenyl)(hydroxy)methyl)benzoate (**27**, 142 mg, 0.324 mmol) in methanol (4 mL) and the mixture was stirred at 50 °C for 12 hours. The mixture was concentrated *in vacuo* and partitioned between aqueous citric acid (10% (w/w)) and dichloromethane. The organic layer was dried over Na_2SO_4 and evaporated *in vacuo*. The residue was dissolved in dichloromethane (10 mL) and *N*,*N*-diisopropylethylamine (90 µL, 0.81 mmol), *N*-(3-dimethylaminopropyl)-*N′*-ethylcarbodiimide hydrochloride (71.4 mg, 0.373 mmol) and *N*-(3-aminopropyl)methacrylamide hydrochloride (**8**, 75.3 mg, 0.421 mmol) were added. After stirring at room temperature for 38 hours, the mixture was diluted with dichloromethane (20 mL) and washed with aqueous citric acid (10% (w/w), 20 mL), aqueous saturated $NaHCO_3$ (20 mL), and brine (20 mL). The organic layer was dried over Na_2SO_4 and evaporated under reduced pressure. The residue was purified by flash chromatography (silica, 60 to 100% ethyl acetate in petroleum ether (boiling range 40-60 °C), containing 0.1% (v/v) triethylamine) to give 4-(bis(2,4-dimethoxyphenyl)(hydroxy)methyl)-*N*-(3-methacrylamidopropyl)benzamide (**28**, 69.1 mg, 39%) as a yellow solid. LCMS: 99%, $[M-OH]^+$ = 531 (method E). ^{1}H NMR (400 MHz, $CDCl_3$): δ 7.72 (d, *J*

= 8.1 Hz, 2H), 7.34 (d, *J* = 8.1 Hz, 2H), 6.98 (t, *J* = 6.6 Hz, 1H), 6.86 (d, *J* = 8.6 Hz, 2H), 6.68 (t, *J* = 6.1 Hz, 1H), 6.49 (d, *J* = 2.4 Hz, 2H), 6.39 (dd, *J* = 8.7, 2.5 Hz, 2H), 5.80 (s, 1H), 5.36 (s, 1H), 5.12 (s, 1H), 3.80 (s, 6H), 3.53 (s, 6H), 3.50 (d, *J* = 6.4 Hz, 2H), 3.42 (q, *J* = 6.4 Hz, 2H), 2.00 (s, 3H), 1.76 (p, *J* = 6.1 Hz, 2H).

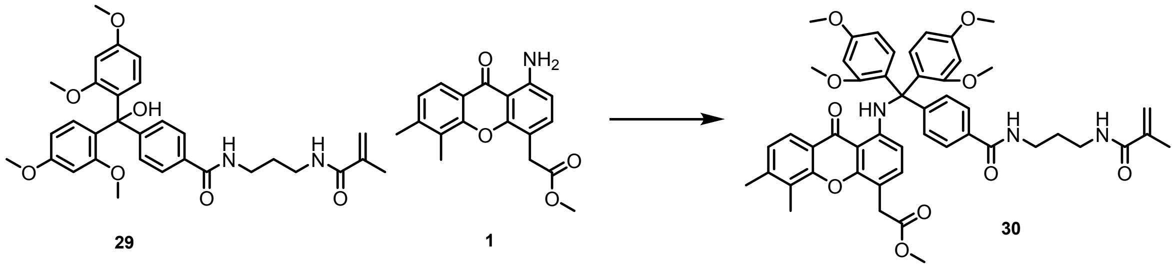

29 **1** **30**

*Methyl2-(1-((bis(2,4-dimethoxyphenyl)(4-((3-methacrylamidopropyl)carbamoyl)phenyl)methyl)-amino)-5,6-dimethyl-9-oxo-9H-xanthen-4-yl)acetate (**30**).*

Under a nitrogen atmosphere, acetyl chloride (0.50 mL, 7.03 mmol) was added to 4-(bis(2,4-dimethoxyphenyl)(hydroxy)methyl)-*N*-(3-methacrylamidopropyl)benzamide (**29**, 60 mg, 0.110 mmol). After stirring for 10 minutes, the dark purple mixture was concentrated *in vacuo*, coevaporated from *n*-heptane (2 x 10 mL) and pressurized with argon. Methyl 2-(1-amino-5,6-dimethyl-9-oxo-9*H*-xanthen-4-yl)acetate (**1**, 34 mg, 0.109 mmol) and pyridine (dry, 1.0 mL, 12.36 mmol) were added. After stirring for 10 minutes, the clear yellow mixture was partitioned between dichloromethane (10 mL) and aqueous saturated $NaHCO_3$ (5 mL). The organic phase was separated using a hydrophobic frit and the filtrate was concentrated under reduced pressure. The residue was purified by column chromatography (alumina (neutral), 50 to 80% ethyl acetate in *n*-heptane (containing 1% (v/v) triethylamine) to give methyl 2-(1-((bis(2,4-dimethoxyphenyl)(4-((3-methacrylamido-propyl)carbamoyl)phenyl)methyl)amino)-5,6-dimethyl-9-oxo-9*H*-xanthen-4-yl)acetate (**30**, 17.4 mg, 19% yield) as a yellow solid. LCMS: 97%, RT = 2.36 min., $[M\text{-}H]^-$ = 840 (method C).

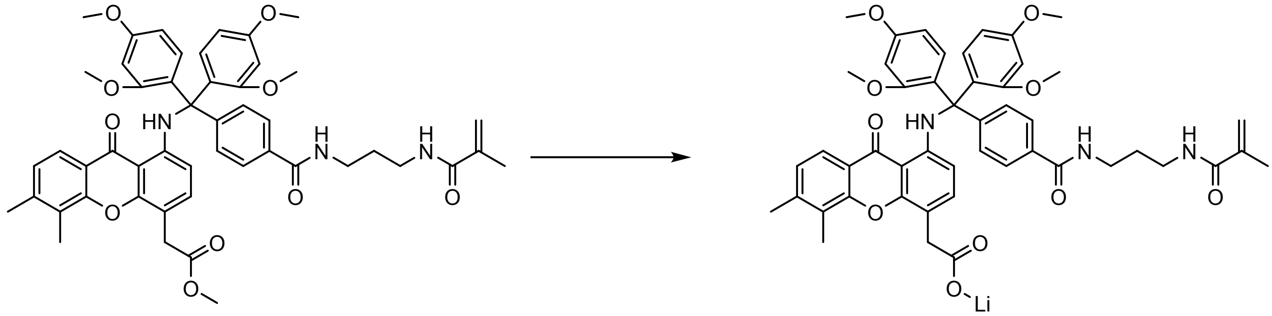

30 **14**

*Lithium2-(1-((bis(2,4-dimethoxyphenyl)(4-((3-ethacrylamidopropyl)carbamoyl)phenyl)methyl)amino)-5,6-dimethyl-9-oxo-9H-xanthen-4-yl)acetate (**14**).*

Under a nitrogen atmosphere, a solution of lithium hydroxide monohydrate (1.8 mg, 0.043 mmol) in water (0.4 mL) was added to a solution of methyl 2-(1-((bis(2,4-dimethoxyphenyl)(4-((3-methacrylamidopropyl)carbamoyl)phenyl)methyl)amino)-5,6-dimethyl-9-oxo-9*H*-xanthen-4-yl)acetate (**30**, 17.4 mg, 0.021 mmol) in tetrahydrofuran (1.0 mL) and the mixture was stirred at room temperature overnight. The mixture was concentrated *in vacuo*, and the residue was coevaporated with dichloromethane and further dried under high vacuum to give lithium 2-(1-((bis(2,4-dimethoxyphenyl)(4-((3-methacrylamidopropyl)carbamoyl)phenyl)methyl)amino)-5,6-dimethyl-9-oxo-9*H*-xanthen-4-yl)acetate (**14**, 10.9 mg, 33% yield). LCMS: 74%, RT = 1.84 min.

Expected mass: 833.9, found $[M\text{-}Li]^-$ = 826 (method C).

^{1}H NMR (400 MHz, DMSO-d_6 + D_2O) δ 10.76 (s, 1H), 7.84 (d, *J* = 8.2 Hz, 1H), 7.69 (d, *J* = 8.3 Hz, 2H), 7.43 – 7.18 (m, 2H), 7.22 (d, *J* = 8.2 Hz, 1H), 6.93 (d, *J* = 8.6 Hz, 1H), 6.63 – 6.35 (m, 4H), 5.99 – 5.70 (m,

1H), 5.67 (s, 1H), 5.37 (s, 1H), 3.74 (s, 6H), 3.41 – 3.13 (m, 12H), 3.08 (q, *J* = 7.3 Hz, 1H), 2.40 (s, 3H), 2.38 (s, 3H), 1.87 (s, 3H), 1.77 – 1.64 (m, 3H), 1.18 – 1.13 (m, 2H).

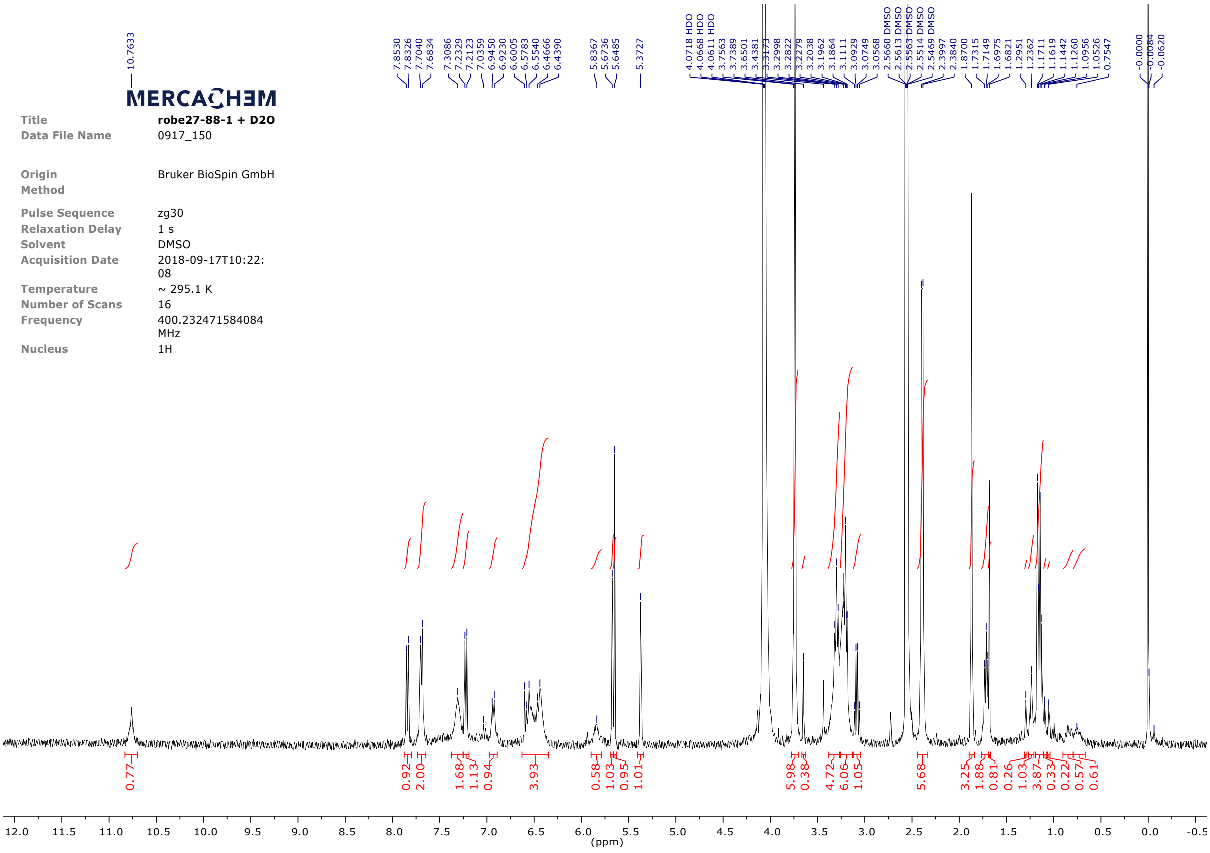

Mixture of atropisomers, contains unknown aliphatic impurities

S2.4:Synthesis of ***15***

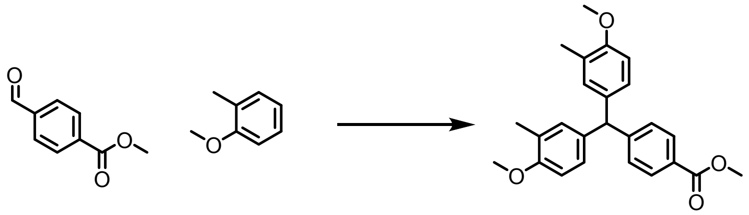

31

*Methyl 4-(bis(4-methoxy-3-methylphenyl)methyl)benzoate (**31**).*

A solution of methyl 4-formyl benzoate (2.46 g, 15.0 mmol) and 2-methylanisol (4.35 mL, 34.5 mmol) in acetic acid (48 mL) was cooled to 0 °C, and concentrated sulfuric acid (7.40 mL) was added dropwise over 10 minutes. The mixture was stirred at room temperature overnight, poured in ice water (250 mL) and extracted with ethyl acetate (3 x 35 mL). Organic layers were combined, washed with water (2 x 25 mL) and brine (25 mL), and dried over $MgSO_4$. The mixture was evaporated under reduced pressure and the residue was purified by flash column chromatography (silica, 0 to 50% ethyl acetate in petroleum ether (boiling range 40-60 °C)) to give methyl 4-(bis(4-methoxy-3-methylphenyl)methyl)benzoate (5.33 g, 91% yield) as a clear viscous oil. ^{1}H NMR (400 MHz, $CDCl_3$) δ

7.94 (d, *J* = 8.3 Hz, 2H), 7.18 (d, *J* = 8.0 Hz, 2H), 6.87 (d, *J* = 2.3 Hz, 2H), 6.83 (dd, *J* = 8.3, 2.4 Hz, 2H), 6.73 (d, *J* = 8.3 Hz, 2H), 5.41 (s, 1H), 3.89 (s, 3H), 3.80 (s, 6H), 2.15 (s, 6H).

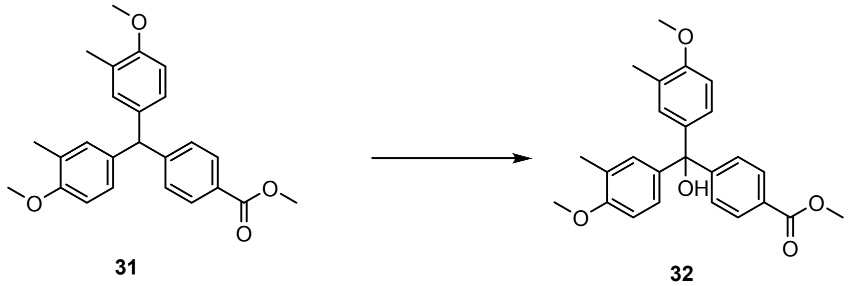

31 **32**

*Methyl 4-(hydroxybis(4-methoxy-3-methylphenyl)methyl)benzoate (**32**).*

A mixture of methyl 4-(bis(4-methoxy-3-methylphenyl)methyl)benzoate (1.33 g, 3.42 mmol) and manganese (IV) dioxide (4.00 g, 46.0 mmol) in toluene (25 mL) was stirred at 110 °C under reflux for 7 days. The reaction mixture was cooled down, diluted with ethyl acetate (40 mL) and filtered through a pad of Celite. The filtrate was concentrated under reduced pressure and the residue was purified by flash column chromatography (silica, 0 to 50% ethyl acetate in petroleum ether (boiling range40-60 °C), containing 0.1% (v/v) triethylamine) to give methyl 4-(hydroxybis(4-methoxy-3-methylphenyl)methyl)benzoate (**32**, 647 mg, 47% yield) as a white foam. ^{1}H NMR (400 MHz, $CDCl_3$): δ 7.96 (d, *J* = 8.2 Hz, 2H), 7.40 (d, *J* = 8.2 Hz, 2H), 7.05 (d, *J* = 2.5 Hz, 2H), 6.95 (dd, *J* = 8.6, 2.5 Hz, 2H), 6.73 (d, *J* = 8.5 Hz, 2H), 3.90 (s, 3H), 3.82 (s, 6H), 2.69 (s, 1H), 2.16 (s, 6H).

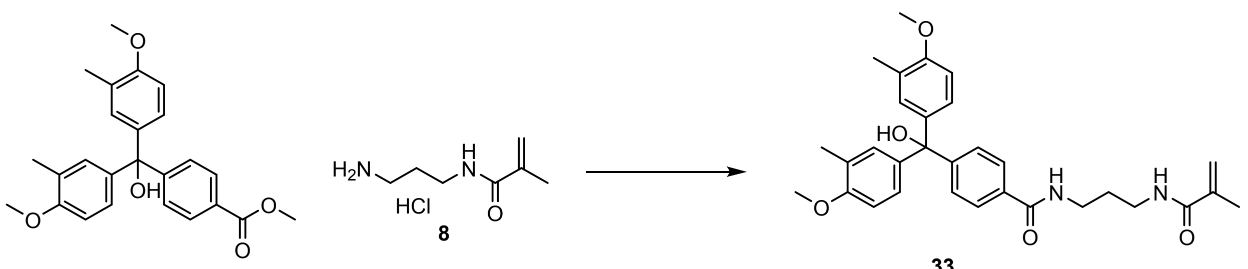

32 **33**

*4-(Hydroxybis(4-methoxy-3-methylphenyl)methyl)-N-(3-methacrylamidopropyl)benzamide(**33**).*

A solution of NaOH (39.0 mg, 0.975 mmol) in water (6 mL) was added to a solution of methyl 4-(hydroxybis(4-methoxy-3-methylphenyl)methyl)benzoate (**32**, 377 mg, 0.929 mmol) in methanol (9 mL) and the mixture was stirred at 50 °C for 12 hours. The mixture was concentrated *in vacuo*. The residue was dissolved in dichloromethane (30 mL) and *N*,*N*-diisopropylethylamine (360 µL, 2.0 mmol), *N*-(3-dimethylaminopropyl)-*N'*-ethylcarbodiimide hydrochloride (210 mg, 1.1 mmol), and *N*-(3-aminopropyl)methacrylamide hydrochloride (220 mg, 2.2 mmol) were added. After stirring at room temperature for 25 hours, the mixture was diluted with dichloromethane (20 mL) and washed with aqueous citric acid (10% (w/w), 20 mL), aqueous saturated $NaHCO_3$ (20 mL), and brine (20 mL). The organic layer was dried over Na_2SO_4 and evaporated under reduced pressure. The residue was purified by flash column chromatography (silica, 60 to 100% ethyl acetate in petroleum ether (boiling range 40-60 °C), containing 0.1% (v/v) triethylamine) to give 4-(bis(2,4-dimethoxyphenyl)(hydroxy)methyl)-*N*-(3-methacrylamidopropyl)benzamide (**33**, 240 mg, 50% yield). LCMS: 99%, $[M-OH]^+$ = 499 (method E). ^{1}H NMR (400 MHz, $CDCl_3$): δ 7.78 (d, *J* = 8.7 Hz, 2H), 7.40 (d, *J* = 8.7 Hz, 2H), 7.12 (t, *J* = 6.3 Hz, 1H), 7.05 (dd, *J* = 2.5, 0.9 Hz, 2H), 6.95 (ddd, *J* = 8.5, 2.5, 0.6 Hz, 2H), 6.72 (d, *J* = 8.5 Hz, 2H), 6.60 (t, *J* = 6.5 Hz, 1H), 5.78 (t, *J* = 1.0 Hz, 1H), 5.37 – 5.34 (m, 1H), 3.82 (s, 6H), 3.50 (q, *J* = 6.3 Hz, 2H), 3.42 (q, *J* = 6.3 Hz, 2H), 2.72 (s, 1H), 2.16 (s, 6H), 2.00 (s, 3H), 1.79 – 1.71 (m, 2H).

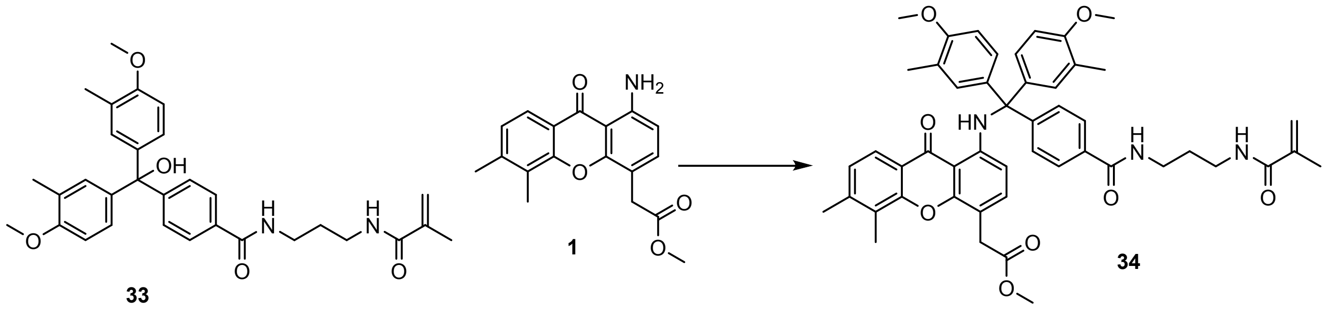

*Methyl2-(1-(((4-((3-methacrylamidopropyl)carbamoyl)phenyl)bis(4-methoxy-3-methylphenyl)-methyl)amino)-5,6-dimethyl-9-oxo-9H-xanthen-4-yl)acetate (**34**).*

Under argon atmosphere, acetyl chloride (0.75 mL, 10.55 mmol) was added to 4-(hydroxybis(4-methoxy-3-methylphenyl)methyl)-*N*-(3-methacrylamidopropyl)benzamide (186 mg, 0.36 mmol). After stirring for 15 minutes, the dark red mixture was concentrated *in vacuo*, coevaporated from *n*-heptane (2 x 10 mL) and pressurized with argon. Methyl 2-(1-amino-5,6-dimethyl-9-oxo-9*H*-xanthen-4-yl)acetate (**1**,112 mg, 0.36 mmol) and pyridine (dry, 1.5 mL, 18.6 mmol) were added. After stirring for 10 minutes, the clear yellow solution was partitioned between dichloromethane (10 mL) and saturated aqueous $NaHCO_3$ (10 mL). The layers were separated using a hydrophobic frit and the organic filtrate was evaporated under reduced pressure. The residue was purified by column chromatography (alumina (neutral), 50 to 80% ethyl acetate in *n*-heptane, containing 1% (v/v) triethylamine) to give methyl 2-(1-(((4-((3-methacrylamidopropyl)-carbamoyl)phenyl)bis(4-methoxy-3-methylphenyl)methyl)amino)-5,6-dimethyl-9-oxo-9*H*-xanthen-4-yl)acetate (**34**, 136.5 mg, 47% yield) as a yellow solid. LCMS: 99%, RT = 2.50 min., $[M-H]^-$ = 808 (method C).

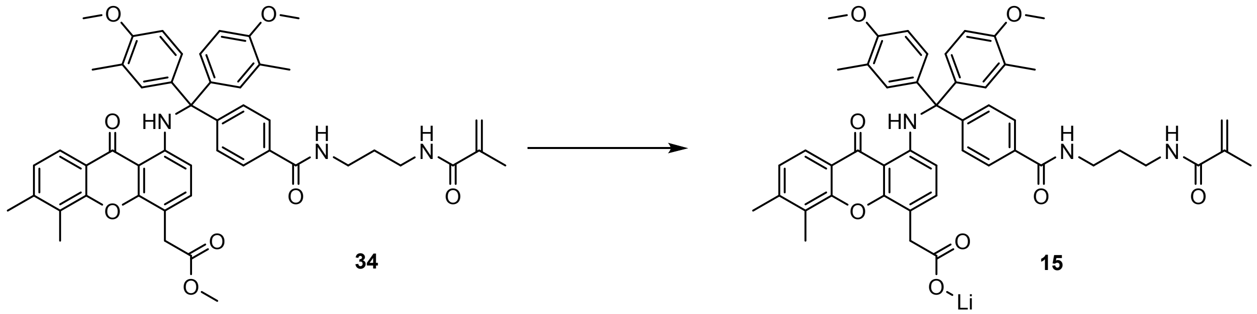

*Lithium2-(1-(((4-((3-methacrylamidopropyl)carbamoyl)phenyl)bis(4-methoxy-3-methylphenyl)methyl)amino)-5,6-dimethyl-9-oxo-9H-xanthen-4-yl)acetate (**15**).*

Under a nitrogen atmosphere, a solution of lithium hydroxide monohydrate (14.3 mg, 0.341 mmol) in water (1.2 mL) was added to a solution of methyl 2-(1-(((4-((3-methacrylamidopropyl)-carbamoyl)phenyl)bis(4-methoxy-3-methylphenyl)methyl)amino)-5,6-dimethyl-9-oxo-9*H*-xanthen-4-yl)acetate (136.5 mg, 0.169 mmol) in tetrahydrofuran (3.0 mL) and the mixture was stirred at room temperature overnight. The mixture was evaporated under reduced pressure and the residue was stirred in dichloromethane (10 mL) for 30 minutes. The turbid mixture was passed through a phase-separator and filtered over Celite. The filtrate was concentrated under reduced pressure and the residue was triturated in diethyl ether. After filtration, the residue was washed with diethyl ether and dried under high vacuum to give lithium 2-(1-(((4-((3-methacrylamidopropyl)carbamoyl)phenyl)bis(4-methoxy-3-methylphenyl)methyl)amino)-5,6-dimethyl-9-oxo-9*H*-xanthen-4-yl)acetate (**15**, 66.9 mg, 44%) as a yellow solid. LCMS: 88%, RT = 3.14 min.

Expected mass: 801.8. found $[M-Li]^-$ = 794 (method D).

^{1}H NMR (400 MHz, DMSO-d_6 + D_2O) δ 11.10 (s, 1H), 7.88 (d, *J* = 8.1 Hz, 1H), 7.76 (d, *J* = 8.4 Hz, 2H), 7.43 (d, *J* = 8.2 Hz, 2H), 7.24 (d, *J* = 8.3 Hz, 1H), 7.14 (d, *J* = 8.6 Hz, 2H), 7.08 (s, 2H), 6.99 (d, *J* = 8.8 Hz,

1H), 6.91 (d, *J* = 8.8 Hz, 2H), 5.80 (d, *J* = 8.6 Hz, 1H), 5.65 (s, 1H), 5.34 (s, 1H), 3.77 (s, 6H), 3.32 (s, 2H), 3.26 (t, *J* = 6.9 Hz, 2H), 3.17 (d, *J* = 13.8 Hz, 2H), 2.41 (s, 3H), 2.39 (s, 3H), 2.07 (s, 6H), 1.85 (s, 3H), 1.80 – 1.63 (m, 2H).

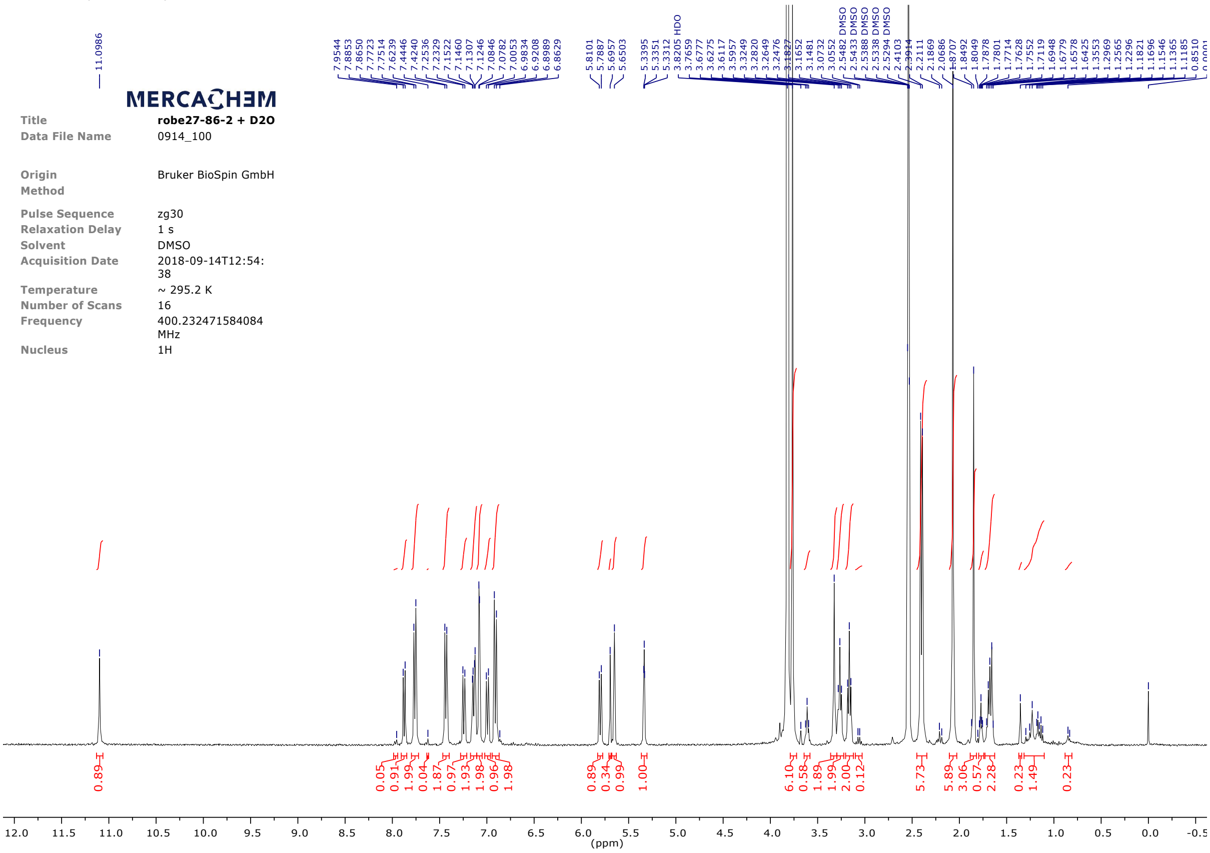

Contains 1.8 %(w/w) DCM, 1.3 %(w/w) THF, and traces of unknown aliphatic impurities

*S2.5: Synthesis of **16***

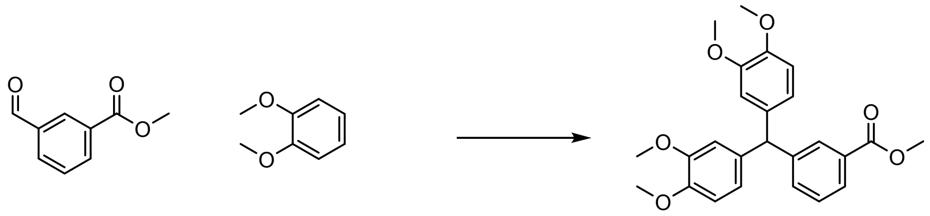

35

*Methyl 3-(bis(3,4-dimethoxyphenyl)methyl)benzoate (**35**).*

A solution of methyl 4-formyl benzoate (492 mg, 3.0 mmol) and 1,2-dimethoxybenzene (0.88 mL, 6.9 mmol) in acetic acid (10 mL) was cooled to 0 °C, and concentrated sulfuric acid (1.5 mL) was added dropwise over 5 minutes. The mixture was stirred at room temperature overnight, poured in ice water (50 mL) and extracted with ethyl acetate (3 x 10 mL). The combined organic layers were washed with water (2 x 10 mL) and brine (10 mL), and dried over $MgSO_4$. After filtration, the filtrate was evaporated under reduced pressure and the residue was purified by flash column chromatography (silica, 0 to 50% ethyl acetate in petroleum ether (40-60 °C)) to give methyl 3-(bis(3,4-dimethoxyphenyl)-methyl)benzoate (1.11 g, 87% yield) as a white solid. ^1H NMR (400 MHz, $CDCl_3$): δ 7.90 (d, *J* = 7.6 Hz, 1H), 7.85 (d, *J* = 1.8 Hz, 1H), 7.36 (t, *J* = 7.7 Hz, 1H), 7.30 (dd, *J* = 7.8, 1.7 Hz, 1H), 6.79 (d, *J* = 8.2 Hz, 2H), 6.65 (d, *J* = 2.0 Hz, 2H), 6.58 (dd, *J* = 8.3, 2.0 Hz, 2H), 5.49 (s, 1H), 3.88 (s, 3H), 3.86 (s, 6H), 3.76 (s, 6H).

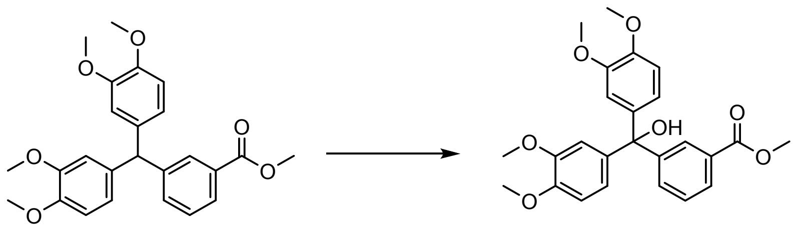

35 **36**

*Methyl 3-(bis(3,4-dimethoxyphenyl)(hydroxy)methyl)benzoate (**36**).*

A mixture of methyl 3-(bis(3,4-dimethoxyphenyl)methyl)benzoate (4.39 g, 10.4 mmol) and manganese (IV) dioxide (10.8 g, 124 mmol) in toluene (80 mL) was stirred at 110 °under reflux C for 49 hours. The reaction mixture was cooled down, diluted with ethyl acetate (200 mL) and filtered through a pad of Celite. The filtrate was concentrated under reduced pressure and the residue was purified by flash column chromatography (silica, 0 to 50% ethyl acetate in petroleum ether (boiling range 40-60 °C), containing 0.1% (v/v) triethylamine) to give methyl 3-(bis(3,4-dimethoxyphenyl)(hydroxy)methyl)benzoate (**36**, 2.98 g, 65% yield). ^{1}H NMR (400 MHz, $CDCl_3$): δ 8.07 (t, J = 1.8 Hz, 1H), 7.96 (dt, J = 7.6, 1.4 Hz, 1H), 7.47 (ddd, J = 7.9, 2.0, 1.3 Hz, 1H), 7.38 (t, J = 7.7 Hz, 1H), 6.90 (d, J = 2.2 Hz, 2H), 6.78 (d, J = 8.4 Hz, 2H), 6.64 (dd, J = 8.4, 2.2 Hz, 2H), 3.88 (s, 3H), 3.88 (s, 6H), 3.77 (s, 6H), 2.81 (s, 1H).

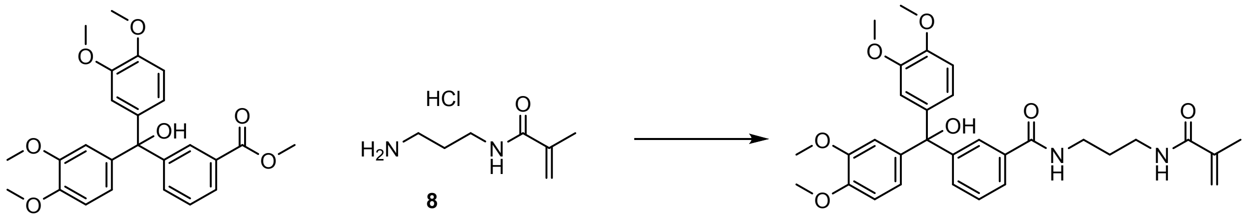

36 **8** **37**

*3-(Bis(3,4-dimethoxyphenyl)(hydroxy)methyl)-N-(3-methacrylamidopropyl)benzamide (**37**).*

A solution of NaOH (0.66 g, 16.4 mmol) in water (80 mL) was added to a solution of methyl 3-(bis(3,4-dimethoxyphenyl)(hydroxy)methyl)benzoate (**36**, 3.15 g, 7.18 mmol) in methanol (120 mL) and the mixture was stirred at 50 °C for 12 hours. The mixture was concentrated *in vacuo* and partitioned between aqueous citric acid (10% (w/w)) and dichloromethane. The organic layer was dried over Na_2SO_4 and evaporated *in vacuo*. The residue was dissolved in dichloromethane (90 mL) and *N*,*N*-diisopropylethylamine (3.1 mL, 18.0 mmol), *N*-(3-dimethylaminopropyl)-*N*′-ethylcarbodiimide hydrochloride (1.60 g, 8.3 mmol) and *N*-(3-aminopropyl)methacrylamide hydrochloride (**8**, 1.70 g, 9.4 mmol) were added. After stirring at room temperature for 34 hours, the mixture was diluted with dichloromethane (100 mL) and washed with aqueous citric acid (10% (w/w), 100 mL), aqueous saturated $NaHCO_3$ (100 mL) and brine (100 mL). The organic layer was dried over Na_2SO_4 and evaporated under reduced pressure, and the residue was purified by flash column chromatography (silica, 60 to 100% ethyl acetate in petroleum ether (40-60 °C), containing 0.1% (v/v) triethylamine) to give 3-(bis(3,4-dimethoxyphenyl)(hydroxy)methyl)-*N*-(3-methacrylamidopropyl)benzamide (**37**, 2.03 g, 51% yield). LCMS: 99%, $[M\text{-}OH]^+$ = 531 (method E). ^{1}H NMR (400 MHz, $CDCl_3$): δ 7.98 (t, J = 1.7 Hz, 1H), 7.78 – 7.75 (m, 1H), 7.36 (t, J = 7.7 Hz, 1H), 7.29 (dt, J = 8.0, 1.5 Hz, 1H), 7.14 (t, J = 6.3 Hz, 1H), 6.93 (d, J = 2.2 Hz, 2H), 6.76 (d, J = 8.4 Hz, 2H), 6.63 (dd, J = 8.4, 2.2 Hz, 2H), 6.62 (t, 1H), 5.77 (t, J = 1.0 Hz, 1H), 5.36 – 5.33 (m, 1H), 3.86 (s, 6H), 3.76 (s, 6H), 3.45 (q, J = 6.2 Hz, 2H), 3.38 (q, J = 6.3 Hz, 2H), 3.23 (s, 1H), 1.98 (s, 3H), 1.79 – 1.63 (m, 2H).

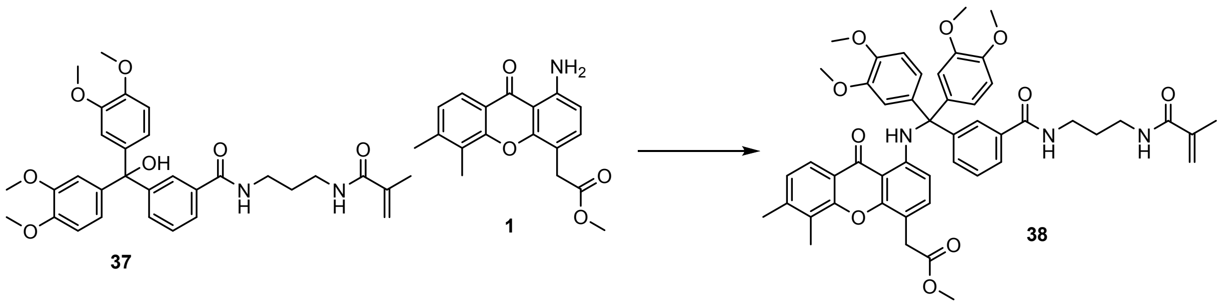

*Methyl2-(1-((bis(3,4-dimethoxyphenyl)(3-((3-methacrylamidopropyl)carbamoyl)phenyl)methyl)-amino)-5,6-dimethyl-9-oxo-9H-xanthen-4-yl)acetate (**38**).*

Under argon atmosphere, acetyl chloride (0.50 mL, 7.03 mmol) was added to 3-(bis(3,4-dimethoxyphenyl)(hydroxy)methyl)-*N*-(3-methacrylamidopropyl)benzamide (108.7 mg, 0.198 mmol). After 15 minutes of stirring, the dark purple reaction mixture was concentrated *in vacuo*, coevaporated from *n*-heptane (2 x 5 mL) and pressurized with argon. Methyl 2-(1-amino-5,6-dimethyl-9-oxo-9*H*-xanthen-4-yl)acetate (**1**, 62 mg, 0.198 mmol) and pyridine (dry, 1.0 mL, 12.4 mmol) were added. After stirring for 15 minutes, the clear yellow mixture was partitioned between dichloromethane (10 mL) and saturated aqueous $NaHCO_3$ (5 mL). The layers were separated using a phase-separator and the organic filtrate was evaporated under reduced pressure. The residue was purified by column chromatography (alumina (neutral), 33 to 100% ethyl acetate in *n*-heptane, containing 1% (v/v) triethylamine) to give methyl 2-(1-((bis(3,4-dimethoxyphenyl)(3-((3-methacrylamidopropyl)carbamoyl)phenyl)methyl)amino)-5,6-dimethyl-9-oxo-9*H*-xanthen-4-yl)acetate (**38**, 44.8 mg, 27% yield) as a yellow solid. LCMS: 93%, RT = 2.25 min., $[M-H]^-$ = 840 (method C).

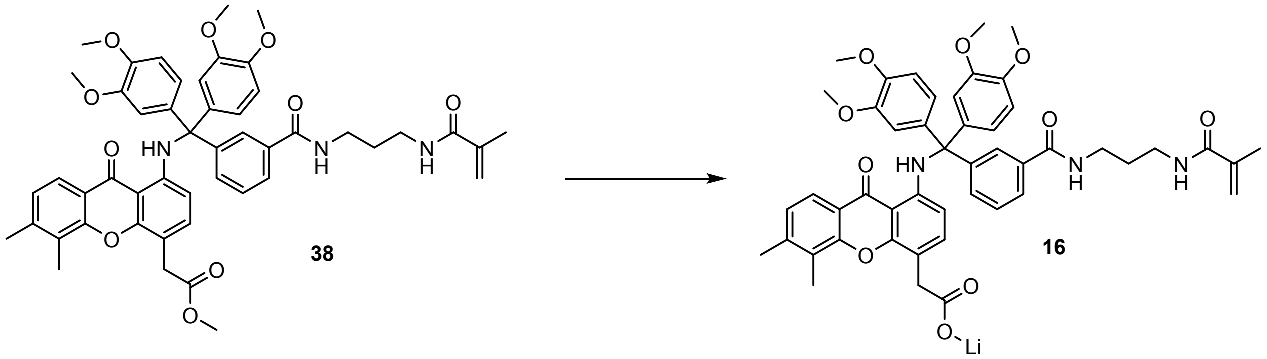

*Lithium2-(1-((bis(3,4-dimethoxyphenyl)(3-((3-methacrylamidopropyl)carbamoyl)phenyl)-methyl)amino)-5,6-dimethyl-9-oxo-9H-xanthen-4-yl)acetate (**16**).*

Under a nitrogen atmosphere, a solution of lithium hydroxide in water (0.135 M, 0.4 mL, 0.054 mmol) was added to a solution of methyl 2-(1-(((4-((3-methacrylamidopropyl)carbamoyl)phenyl)bis(4-methoxy-3-methylphenyl)methyl)amino)-5,6-dimethyl-9-oxo-9*H*-xanthen-4-yl)acetate (44.8 mg, 0.053 mmol) in tetrahydrofuran (1.0 mL) and the mixture was stirred at room temperature overnight. Extra tetrahydrofuran (0.5 mL) and a solution of lithium hydroxide in water (0.135 M, 0.4 mL, 0.054 mmol) were added and the mixture was stirred for another 7 hours at room temperature and concentrated under reduced pressure. The aqueous residue was diluted with water (1.0 mL) and acetonitrile (2.0 mL), and purified by basic preparative MPLC (Waters XSelect CSH C18 (145x25 mm, 10µ), linear gradient: t=0 min 5% B; t=1 min 5% B; t=17 min 50% B; t=23 min 100% B; detection: ELSD). The combined product fractions were lyophilized to give lithium 2-(1-((bis(3,4-dimethoxyphenyl)(3-((3-methacrylamidopropyl)carbamoyl)phenyl)methyl)amino)-5,6-dimethyl-9-oxo-9*H*-xanthen-4-yl)acetate (**16**, 18.6 mg, 42% yield) as a yellow solid. LCMS: 98%, RT = 2.91 min.
Expected mass: 833.9, found $[M-Li]^-$ = 826 (method D).

^{1}H NMR (400 MHz, DMSO-d_6) δ 11.21 (s, 1H), 8.49 (t, *J* = 5.8 Hz, 1H), 7.96 – 7.86 (m, 3H), 7.73 (d, *J* = 7.6 Hz, 1H), 7.53 – 7.39 (m, 2H), 7.25 (d, *J* = 8.3 Hz, 1H), 7.09 (d, *J* = 8.8 Hz, 1H), 6.94 (d, *J* = 8.6 Hz, 2H), 6.90 – 6.83 (m, 4H), 5.85 (d, *J* = 8.6 Hz, 1H), 5.62 (s, 1H), 5.29 (s, 1H), 3.74 (s, 6H), 3.64 – 3.51 (m, 8H), 3.25 – 3.18 (m, 2H), 3.12 (q, *J* = 6.7 Hz, 2H), 2.41 (s, 3H), 2.34 (s, 3H), 1.82 (s, 3H), 1.64 (p, *J* = 6.9 Hz, 2H).

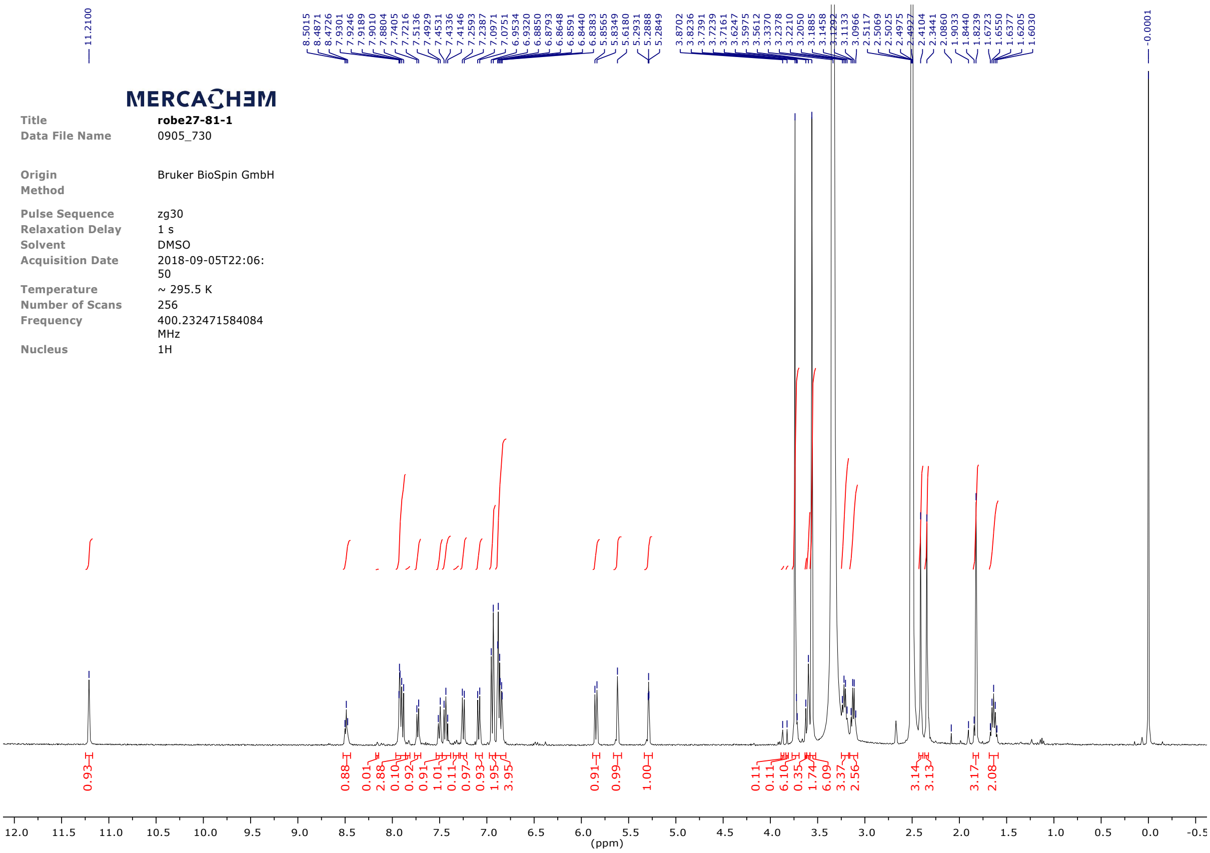

S2.6: Synthesis of 17

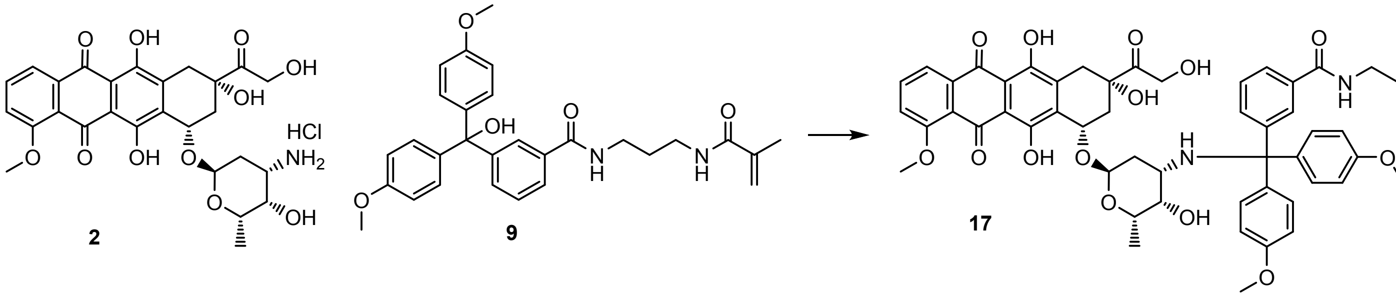

*3-((((2S,3S,4S,6R)-3-hydroxy-2-methyl-6-(((1S,3S)-3,5,12-trihydroxy-3-(2-hydroxyacetyl)-10-methoxy-6,11-dioxo-1,2,3,4,6,11-hexahydrotetracen-1-yl)oxy)tetrahydro-2H-pyran-4-yl)amino)bis(4-methoxyphenyl)methyl)-N-(3-methacrylamidopropyl)benzamide (**17**).*

(3-(Chlorobis(4-methoxyphenyl)methyl)-*N*-(3-methacrylamidopropyl)benzamide (**9**, 0.041 mmol) was dissolved in dry dichloromethane (1.0 mL) and added to a solution of doxorubicin hydrochloride (**2**, 25 mg, 0.043 mmol) and *N*,*N*-diisopropylethylamine (21.4 µl, 0.123 mmol) in dry pyridine (1.0 mL). The obtained mixture was stirred at room temperature for 2 days. Then, an extra quantity of *N*,*N*-diisopropylethylamine (25 µl, 0.144 mmol) and freshly prepared 3-(chlorobis(4-methoxyphenyl)methyl)-*N*-(3-methacrylamidopropyl)benzamide (0.051 mmol) in dichloromethane (dry, 1.0 mL) were added and the mixture was stirred for 20 hours. Next, an extra quantity of freshly

prepared 3-(chlorobis(4-methoxyphenyl)methyl)-*N*-(3-methacrylamidopropyl)benzamide (0.051 mmol) in dichloromethane (dry, 0.5 mL) was added and the mixture was stirred for 20 hours and concentrated under reduced pressure. The residue was purified by basic preparative MPLC (Waters XSelect CSH C18 (145x25 mm, 10µ), linear gradient: t=0 min 20% B; t=1 min 20% B; t=17 min 70% B; t=18 min 100% B; detection: ELSD). The combined product fractions were lyophilized to give 3-((((2*S*,3*S*,4*S*,6*R*)-3-hydroxy-2-methyl-6-(((1*S*,3*S*)-3,5,12-trihydroxy-3-(2-hydroxyacetyl)-10-methoxy-6,11-dioxo-1,2,3,4,6,11-hexahydrotetracen-1-yl)oxy)tetrahydro-2*H*-pyran-4-yl)amino)bis(4-methoxyphenyl)methyl)-*N*-(3-methacrylamidopropyl)benzamide (**17**, 30 mg, 68% yield) as a red solid.
LCMS: 89%, RT = 4.05 min.
Expected mass: 1013.3, found $[M-H]^-$ = 1012 (method D).
^{1}H NMR (400 MHz, DMSO-d_6) δ 8.31 (t, *J* = 5.7 Hz, 1H), 8.00 – 7.88 (m, 4H), 7.70 (dd, *J* = 6.6, 3.4 Hz, 1H), 7.61 – 7.49 (m, 2H), 7.31 – 7.20 (m, 5H), 7.11 – 6.81 (m, 1H), 6.77 – 6.62 (m, 4H), 5.62 (s, 1H), 5.33 – 5.25 (m, 2H), 4.97 (s, 1H), 4.91 – 4.84 (m, 1H), 4.81 (t, *J* = 6.0 Hz, 1H), 4.50 – 4.40 (m, 3H), 4.02 (s, 3H), 3.75 – 3.70 (m, 1H), 3.69 – 3.61 (m, 1H), 3.60 (s, 3H), 3.53 (s, 3H), 3.20 – 3.06 (m, 5H), 3.04 – 2.82 (m, 3H), 2.71 – 2.61 (m, 1H), 2.27 (d, *J* = 8.7 Hz, 1H), 2.11 – 1.98 (m, 2H), 1.83 (s, 3H), 1.64 – 1.54 (m, 3H), 0.94 (d, *J* = 6.5 Hz, 3H).

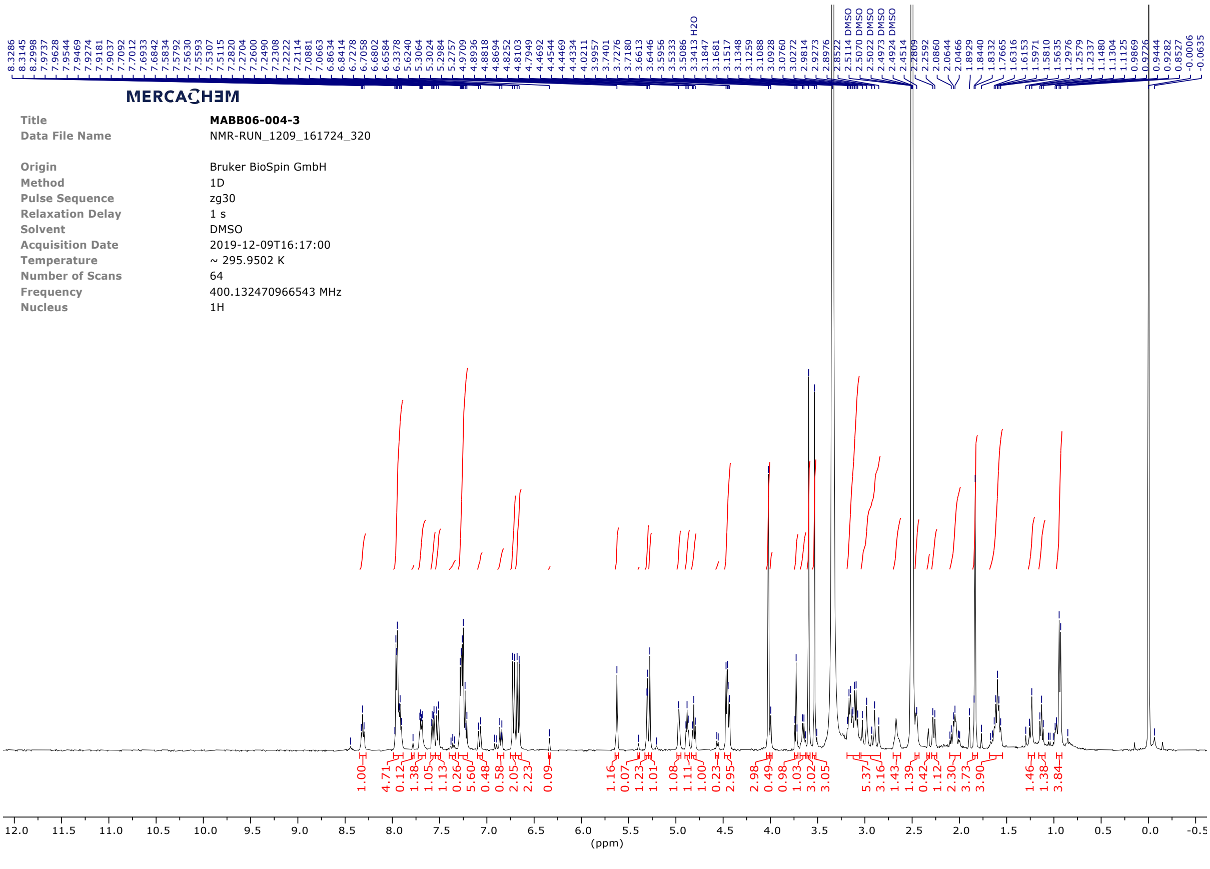

One signal (1H) coincides with DMSO signal. Contains unknown impurities.

S2.7: Synthesis of ***18***

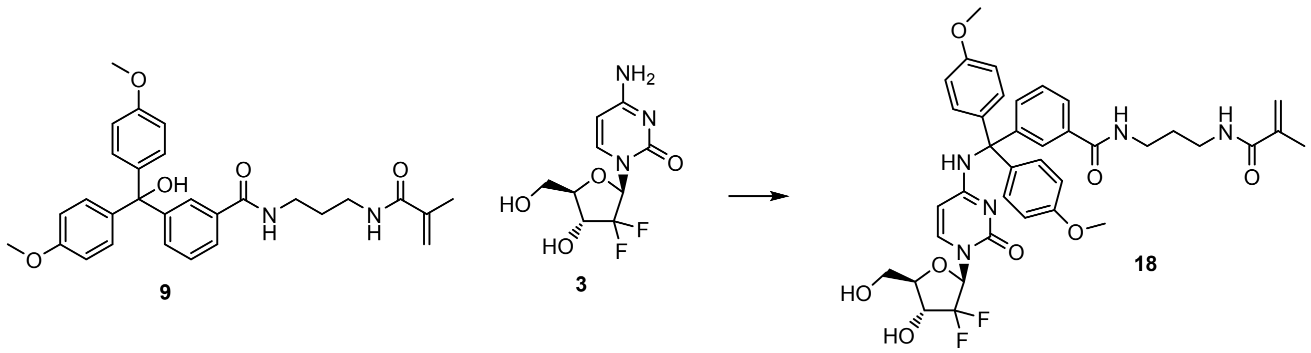

*3-(((1-((2R,4R,5R)-3,3-Difluoro-4-hydroxy-5-(hydroxymethyl)tetrahydrofuran-2-yl)-2-oxo-1,2-dihydropyrimidin-4-yl)amino)bis(4-methoxyphenyl)methyl)-N-(3-methacrylamidopropyl)benzamide (**18**).*

A solution of 3-(chlorobis(4-methoxyphenyl)methyl)-*N*-(3-methacrylamidopropyl)benzamide (**9**, 0.41 mmol) in dichloromethane (dry, 4.0 mL) was added via syringe to a an ice cooled solution of Gemcitabine (**3**, 124 mg, 0.47 mmol, coevaporated with pyridine (dry, 3 x 4.0 mL)) in pyridine (dry, 4.0 mL). After stirring at room temperature for 4 hours, the reaction mixture was concentrated under reduced pressure and the residue was purified by basic preparative MPLC (Waters XSelect CSH C18 (145x25 mm, 10µ), linear gradient: t=0 min 20% B; t=1 min 20% B; t=17 min 60% B; t=18 min 100% B; detection: ELSD). The combined product fractions were concentrated under reduced pressure, dissolved in a mixture of acetonitrile and methanol (1:1 (v/v), 20 mL), dried over Na_2SO_4 and concentrated under reduced pressure. The residue was triturated from diethyl ether, washed with pentane, and air dried (on the filter) to yield 3-(((1-((2*R*,4*R*,5*R*)-3,3-difluoro-4-hydroxy-5-(hydroxymethyl)tetrahydrofuran-2-yl)-2-oxo-1,2-dihydropyrimidin-4-yl)amino)bis(4-methoxyphenyl)-methyl)-*N*-(3-methacrylamidopropyl)benzamide (**18**, 51 mg, 17% yield) as a white solid. LCMS: 98%, RT = 3.26 min.

Expected mass: 733.7, found$[M-H]^-$ = 732 (method D).

^1H NMR (400 MHz, DMSO-d_6) δ 8.62 (s, 1H), 8.44 (t, *J* = 5.8 Hz, 1H), 7.95 (t, *J* = 5.8 Hz, 1H), 7.78 (s, 1H), 7.66 (d, *J* = 6.8 Hz, 1H), 7.60 (d, *J* = 7.7 Hz, 1H), 7.40 – 7.30 (m, 2H), 7.18 – 7.08 (m, 4H), 6.95 – 6.83 (m, 4H), 6.29 (d, *J* = 7.7 Hz, 1H), 6.22 (d, *J* = 6.6 Hz, 1H), 5.97 (t, *J* = 8.5 Hz, 1H), 5.64 (s, 1H), 5.31 (t, *J* = 1.7 Hz, 1H), 5.21 – 5.04 (m, 1H), 4.17 – 4.06 (m, 1H), 3.72 (s, 8H), 3.62 – 3.50 (m, 1H), 3.22 (q, *J* = 6.7 Hz, 2H), 3.14 (q, *J* = 6.6 Hz, 2H), 1.84 (s, 3H), 1.65 (p, *J* = 6.9 Hz, 2H).

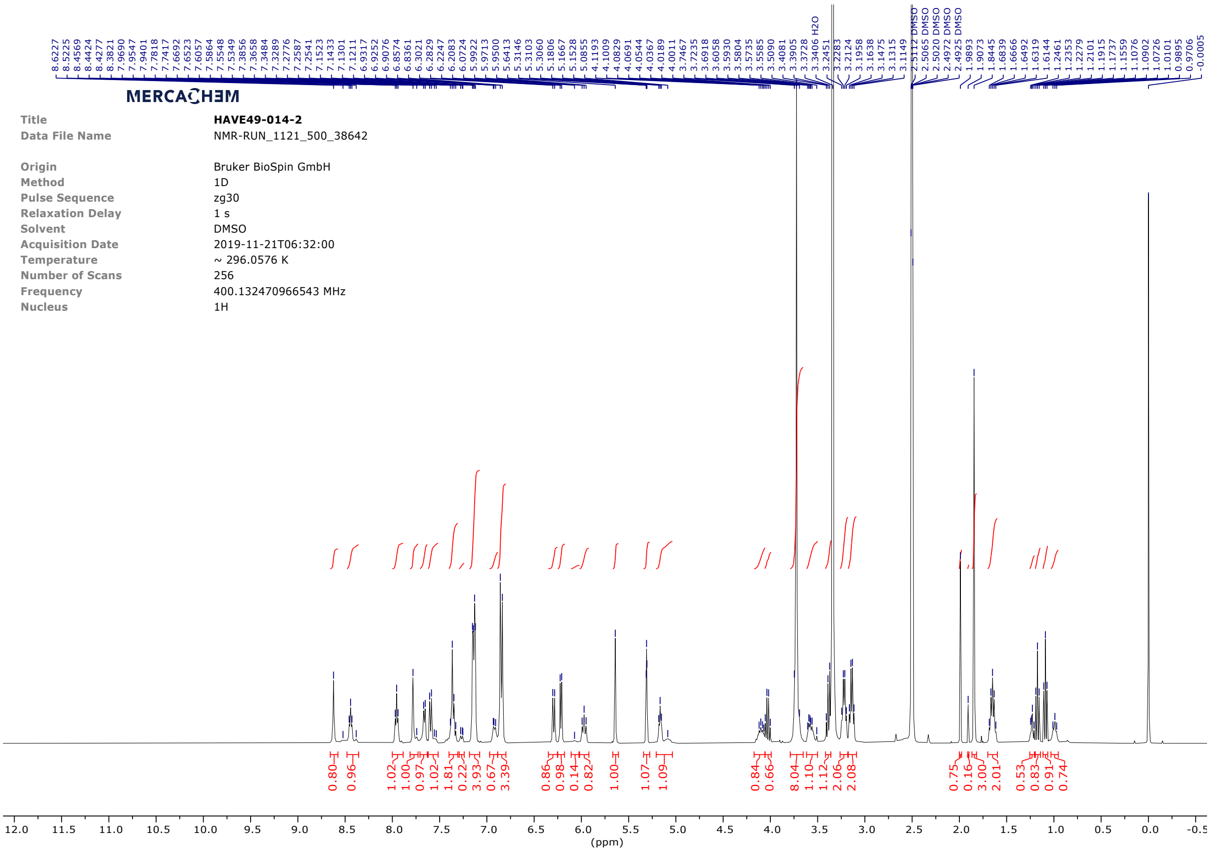

Contains 3.2% (w/w) EtOAc, 1.5% (w/w) Et2O, and unknown aliphatic impurities.

S3: In vitro release protocol and stability control

S3.1 Release of API from API-linker construct

API-linker constructs **12-16**, **17** and **18** were dissolved in DMSO at 1 mg/mL. This was then further diluted 10x in 20 mM sodium phosphate buffer 130 mM NaCl pH 7.4, 100 mM sodium citrate buffer 50 mM NaCl pH 6.5, 20 mM ammonium acetate 130 mM NaCl pH 5.0 or if needed 100 mM sodium phosphate buffer 50 mM NaCl pH 3.0. These dilutions were incubated at 37°C in the sample tray and at different timepoints, the concentration of free API **1**, **2**, or **3** was measured on a H-class UPLC system (Waters) (Figure S7 and S8)

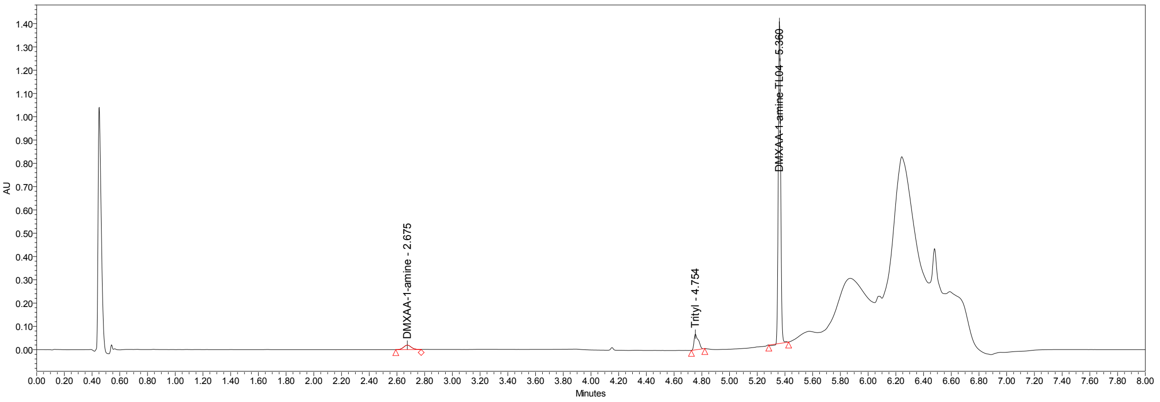

Figure S 7: Chromatogram of 15 at 37°C, pH 7.4 t=0 hours

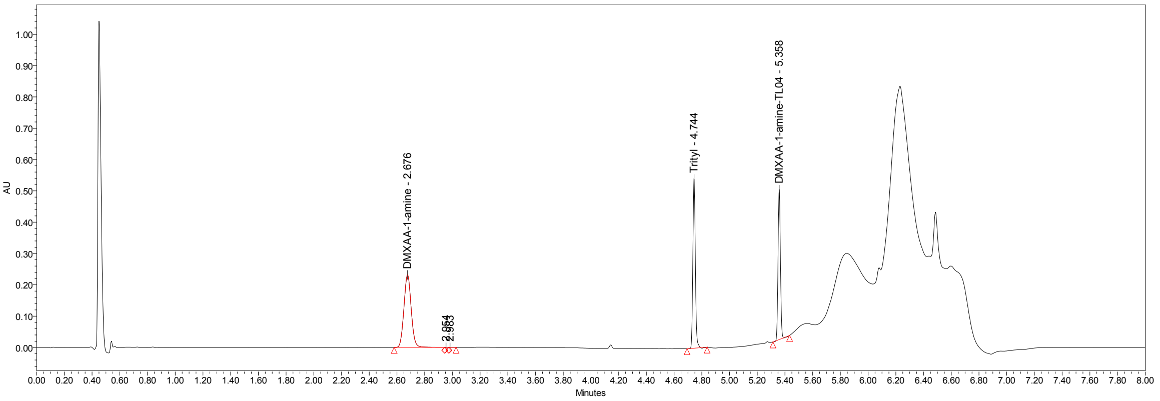

Figure S 8: Chromatogram of 15 at 37°C, pH 7.4, t= 12 hours

1: an Acquity UPLC CSH C18 1.7µm, 2.1 x 100 mm column (Waters) for **12-16** at 236 nm, 2.0 µL injection volume, 30 °C column temperature. Run with the following gradient:

Time (min)	Flow rate (ml/min)	%A	%B
Initial	0.500	70.0	30.0
2.00	0.500	70.0	30.0
5.00	0.500	10.0	90.0
5.50	0.500	10.0	90.0
6.00	0.500	70.0	30.0
8.00	0.500	70.0	30.0

With A: 25 mM Ammonium acetate buffer pH 5.0 and B: acetonitrile.

2: Acquity HSS T3 1.7 µm, 50 x 2.1 mm column for **18** at 275 nm, 5.0 µL injection volume, 30 °C column temperature. Run with the following gradient:

Time (min)	Flow rate (mL/min)	% A	% B
Initial	0.500	97.0	3.0
1.50	0.500	97.0	3.0
2.50	0.500	50.0	50.0
3.80	0.500	50.0	50.0
4.00	0.500	97.0	3.0
5.00	0.500	97.0	3.0

With A: phosphate buffer pH 2.5 and B: methanol.

3: Acquity BEH C18 1.7 µm, 50 x 2.1 mm for **18** at 233 nm, 2.0 µL injection volume, 30 °C column temperature. Run with the following gradient:

Time (min)	Flow rate (ml/min)	%A	%B
Initial	0.500	95.0	5.0
4.50	0.500	2.0	98.0
5.00	0.500	2.0	98.0
5.50	0.500	95.0	5.0
8.00	0.500	95.0	5.0

With A: 10 mM ammonium bicarbonate pH 9.6 and B: acetonitrile.

Measured concentrations were plotted using GraphPad Prism 9 and fitted based on first order kinetics. All fitted lines complied with an R^2>0.95. This gave the half-life and plateau when this was not reached within the measured timeframe.

S3.2 Release of API from CCPM

CCPM-NPs containing **1**, **2** and **3** crosslinked in the core and dispersed in phosphate buffer pH 7.4 at 20 mg/mL polymer and 1 mg/mL API equivalent were diluted 10x in sodium phosphate 20mM NaCl 130 mM buffer pH 7.4, sodium citrate 100mM 50 mM NaCl buffer pH 6.5, ammonium acetate 20 mM NaCl 130 mM pH 5.0 or if needed sodium phosphate 100 mM NaCl 50 mM pH 3.0. These dilutions were incubated at 37°C and at different timepoints, sample were drawn in which the concentration of free API **1**, **2**, or **3** was measured as described in S3.1.

S4: CCPM Formulation protocol

Block copolymer was obtained from Cristal Therapeutics, with the following properties:

**Molecular weight**	22 KDa (NMR)
**PEG-initiator used**	$(OMe\text{-}PEG_{5000})_2$ABCPA
**Lac_1-Lac_2 ratio**	45:55 (NMR)
**Cloudpoint**	8.9 °C (DLS)
**Methacrylation degree**	8.9% (NMR)

Core-crosslinked polymeric micelles containing different API-linker constructs **12-16, 17** and **18** were prepared using the fast-heating method.[63,73] In brief, for a 2 mL batch, an ice-cooled aqueous solution of methacrylated mPEG-b-pHPMAmLac$_m$ block copolymer (1.66 mL of 24.1 mg/mL polymer in phosphate buffer pH 7.4) was mixed with TEMED (0.05 mL of 120 mg/mL stock with pH adjusted to 7.4 using phosphoric acid). subsequently, the API-linkers (10 mg/mL drug equiv., in 0.2 mL DMSO) were added, followed by heating while stirring vigorously to form polymeric micelles. KPS (0.09 mL of 30 mg/mL stock in phosphate buffer) was added to the micellar dispersion. The polymeric micelles were covalently stabilised by polymerisation of the methacrylate moieties on the block copolymer under a N_2 atmosphere at room temperature for 1 h to yield drug-containing CCPMs. Subsequently, the CCPMs were filtered through 0.2 µm cellulose membrane filters to remove potentially formed aggregates. Before measuring size, small impurities were removed using ViVaspin centrifugal concentrators with a 10kDa MWCO regenerated cellulose membrane. Size of all particles were confirmed to be between 55 and 70 nm.

Particle analysis:

	DLS: size (nm)	PDI
CCPM **12**	65.2 ± 0.4	0.063 ± 0.018
CCPM **13**	65.5 ± 0.4	0.056 ± 0.016
CCPM **14**	67.5 ± 1.2	0.082 ± 0.004
CCPM **15**	64.0 ± 1.1	0.069 ± 0.008
CCPM **16**	65.2 ± 0.4	0.063 ± 0.017
CCPM **17**	59.8 ± 1.4	0.069 ± 0.020
CCPM **18**	62.23 ± 0.2	0.044 ± 0.014
